# Rapid dynamics of general transcription factor TFIIB binding during preinitiation complex assembly revealed by single-molecule analysis

**DOI:** 10.1101/gad.285395.116

**Published:** 2016-09-15

**Authors:** Zhengjian Zhang, Brian P. English, Jonathan B. Grimm, Stephanie A. Kazane, Wenxin Hu, Albert Tsai, Carla Inouye, Changjiang You, Jacob Piehler, Peter G. Schultz, Luke D. Lavis, Andrey Revyakin, Robert Tjian

**Affiliations:** 1 Transcription Imaging Consortium, Janelia Research Campus, Howard Hughes Medical Institute, Ashburn, Virginia 20147, USA;; 2 Department of Chemistry, The Scripps Research Institute, La Jolla, California 92037 USA;; 3 Howard Hughes Medical Institute, Department of Molecular and Cell Biology, University of California at Berkeley, Berkeley, California 94720, USA;; 4 Li Ka Shing Center for Biomedical and Health Sciences, University of California at Berkeley, Berkeley, California 94720, USA;; 5 Department of Biology, University of Osnabrück, 49076 Osnabrück, Germany;; 6 Department of Molecular and Cell Biology, University of Leicester, Leicester LE1 9HN, United Kingdom

**Keywords:** single-molecule, fluorescence imaging, dynamic analysis, transcription, preinitiation complex, in vitro reconstitution

## Abstract

Transcription of protein-encoding genes in eukaryotic cells requires the coordinated action of multiple general transcription factors (GTFs) and RNA polymerase II (Pol II). A “step-wise” preinitiation complex (PIC) assembly model has been suggested based on conventional ensemble biochemical measurements, in which protein factors bind stably to the promoter DNA sequentially to build a functional PIC. However, recent dynamic measurements in live cells suggest that transcription factors mostly interact with chromatin DNA rather transiently. To gain a clearer dynamic picture of PIC assembly, we established an integrated in vitro single-molecule transcription platform reconstituted from highly purified human transcription factors and complemented it by live-cell imaging. Here we performed real-time measurements of the hierarchal promoter-specific binding of TFIID, TFIIA, and TFIIB. Surprisingly, we found that while promoter binding of TFIID and TFIIA is stable, promoter binding by TFIIB is highly transient and dynamic (with an average residence time of 1.5 sec). Stable TFIIB–promoter association and progression beyond this apparent PIC assembly checkpoint control occurs only in the presence of Pol II–TFIIF. This transient-to-stable transition of TFIIB-binding dynamics has gone undetected previously and underscores the advantages of single-molecule assays for revealing the dynamic nature of complex biological reactions.

## Abstract (precis)

In this study, Zhang et al present a single-molecule imaging-based dynamic analysis of human RNA polymerase II preinitiation complex (PIC) assembly. They established an integrated in vitro single-molecule transcription platform reconstituted from highly purified human transcription factors and complemented by live-cell imaging and performed real-time measurements of the hierarchal promoter-specific binding of TFIID, TFIIA, and TFIIB.

Transcription initiation by eukaryotic RNA polymerase II (Pol II) requires the coordinated action of Pol II and at least six general transcription factors (GTFs; i.e., TFIIA, TFIIB, TFIID, TFIIE, TFIIF, and TFIIH) at promoters of protein-encoding genes (Thomas and Chiang 2006). It is generally believed that the specificity of PIC nucleation is achieved primarily by two GTFs, TFIID and TFIIB, both known to have direct DNA-binding activity and modulated by TFIIA. Thus, classical biochemical studies have established a “step-wise” model for the assembly of a Pol II transcription preinitiation complex (PIC) (Buratowski et al. 1989). In this model, the core promoter recognition factor—TFIID, composed of the TATA-binding protein (TBP) and ∼14 TBP-associated factors (TAFs)—is the first factor to bind promoter DNA (Albright and Tjian 2000; Matangkasombut et al. 2004). Notably, TBP can substitute for TFIID to support a “basal” level of transcription but is unable to respond to activators. Binding of TFIID/TBP to the promoter is stabilized by TFIIA followed by the binding of TFIIB, which in turn recruits the Pol II–TFIIF subassembly. TFIIE and TFIIH are the last to join the PIC assembly and are thought to facilitate efficient promoter melting and transcription initiation (Thomas and Chiang 2006). As an alternative to the step-wise model, it has also been proposed that, instead of functioning as discrete components, some GTFs, including TFIIB and Pol II, may join the promoter as part of a stable preassembled “holoenzyme” (Wilson et al. 1996). However, it remains unclear whether such preassembled stable holoenzyme complexes play a functional role in vivo.

These early biochemical studies defined the basic role of each GTF in transcription initiation and have revealed numerous protein–protein and protein–DNA interactions within the PIC (Thomas and Chiang 2006). However, the timing and coordination of these interactions remained elusive. Initial biochemical characterization suggested that transcription initiation may take 30 min to 1 h in vitro (Hawley and Roeder 1987), and similar or longer time scales hold true for transcription initiation in the cell as measured by the latest single-molecule imaging-based technologies (Larson et al. 2013). On the other hand, much shorter residence times have been reported for gene-specific activators (Chen et al. 2014) and the GTF TFIIB in living cells (Chen et al. 2002). It is reasonable to expect that more dynamic interactions occur during in vivo PIC assembly, which likely contributes to regulating this essential multifactor multistep reaction. One critical aspect of cell type regulation is to choose the right promoter in the genome to initiate transcription—a challenge given that typical core promoters are ∼100 base pairs (bp) sparsely imbedded in a human genome of 6 billion base pairs. Here, we focused our analysis on the three initial GTFs critical for the nucleation of PIC assembly—TFIID, TFIIA, and TFIIB—as a starting point for gaining new insights into transcriptional control mechanisms.

The TBP subunit of TFIID recognizes and binds to the TATA box, while TAF1 and TAF2 interact with the Initiator element (Inr), and TAF1 and the TAF6–TAF9 module recognize the downstream core promoter element (DPE) (Juven-Gershon and Kadonaga 2010; He et al. 2016; Louder et al. 2016). Classical “footprinting” assays have indicated that TFIID can protect an extended region spanning from the TATA box to beyond the DPE element on numerous Pol II promoters (Nakajima et al. 1988; Purnell et al. 1994). On the other hand, it has also been noted that highly purified TFIID, when bound to the supercore promoter DNA, fails to protect the TATA box (Cianfrocco et al. 2013). This is likely due to occlusion of the DNA-binding surface of TBP by TAF1 in the context of TFIIA, an inhibition that can be alleviated by the small three-subunit TFIIA complex (Bagby et al. 2000; Cianfrocco et al. 2013). TFIIA may therefore function as a “coactivator” at some promoters (Wu et al. 1998) in a way similar to the TAF subunits of TFIID (Albright and Tjian 2000). TFIIA is thought to recognize no specific sequence in the promoter but mainly contacts the DNA phosphate-ribose backbone in the TBP–TATA–TFIIA ternary complex (Geiger et al. 1996; Tan et al. 1996). Proper binding of TBP to the TATA box or a TATA-like element is likely to be critical for the engagement of TFIIB, which recognizes both a surface on TBP and DNA sequences next to the TATA box (Tsai and Sigler 2000), referred to as the TFIIB response elements (BREs) (Lagrange et al. 1998; Deng and Roberts 2006). TFIIB is a single polypeptide that is evolutionally conserved in archaea and eukaryotes (Werner and Grohmann 2011). TBP and promoter binding is carried out by a domain (amino acid residues 107–316) of TFIIB containing two imperfect repeats of cyclin folds (Tsai and Sigler 2000). The N terminus of TFIIB (amino acid residues 1–75) contains a zinc ribbon domain followed by a flexible region that is thought to directly interact with Pol II to trigger transcription activity (Chen and Hahn 2003; Sainsbury et al. 2013). Thus, TFIIB is the simplest but critical component coordinating promoter recognition and RNA synthesis. Paradoxically, in vivo studies suggested that the half-life of TFIIB–chromatin interactions, on average, is in the range of seconds, which is in contrast to the minutes-long residence times of TBP observed in the same study (Chen et al. 2002). Thus, we were particularly interested to determine how TFIIB is recruited to the promoter, the dynamics of its binding, and how these dynamics might influence or mediate transcription initiation, which occurs on a much longer time scale.

Accurate measurement of the temporal dynamics in the formation of macromolecular assemblies during Pol II transcription initiation poses a considerable challenge for conventional biochemistry because of the intrinsic elementary stochastic interactions of each PIC components. Therefore, we previously established an in vitro single-molecule platform capable of monitoring individual molecular interactions and transcription initiation outcome at immobilized single-DNA templates with sub-second temporal resolution (Revyakin et al. 2012; Zhang et al. 2014) based on a fluorescence colocalization spectroscopy technique (Friedman and Gelles 2015). Here we adapted our single-molecule imaging platform to characterize the dynamics of promoter-specific binding of TFIID, TFIIA, and TFIIB. We found TBP alone to lack sufficient specificity in recognizing and binding to a physiologically relevant length of a TATA-box-containing DNA fragment. This apparent lack of specificity can be rectified by the presence of TAF subunits of the holo–TFIID complex. Most surprisingly, we found that TFIID- and TFIIA-dependent TFIIB binding is transient, with a residence time of ∼1.5 sec, which becomes stabilized only after a specific interaction with Pol II–TFIIF, indicating a transition to a functional PIC. We further confirmed these in vitro findings by live-cell single-molecule imaging. The unexpectedly rapid and transient TFIIB promoter binding and its subsequent stabilization by Pol II–TFIIF provide a more complete picture of mechanisms modulating PIC assembly and reveal how the dynamic behavior of TFIIB may lead to productive transcription in vivo. Our studies also underscore the advantages of superresolution dynamic imaging studies to uncover previously undetected mechanisms regulating complex reactions such as those taking place during Pol II PIC assembly.

## Results

### TFIID but not TBP binds promoter DNA specifically

**Figure 1.**
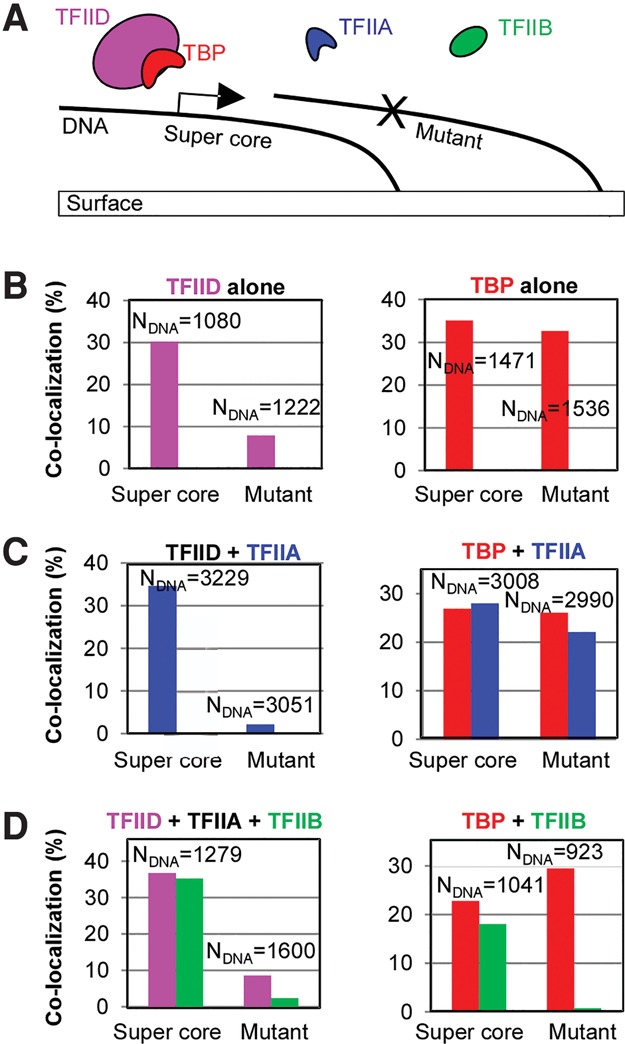
Promoter-specific binding of Pol II GTFs TFIID/TBP, TFIIA, and TFIIB. (*A*) Scheme: Two DNA templates containing either the synthetic supercore promoter or its “null” mutant (Juven-Gershon et al. 2006) were immobilized on the single-molecule imaging surface. The arrow indicates the transcription start site. Transcription factors were incubated, and the DNA binding of the fluorescently labeled molecules was monitored in real time. (*B*–*D*) Colocalization results: The percentage of each DNA template colocalizing with detectable protein signals during a 27-min incubation was plotted. All factors used in each assay are listed with the labeled factors color-coded and unlabeled factors in black. TFIID was used at ∼1 nM and labeled with Atto565-Tris-NTA. TBP was used at 2 nM and labeled with Janelia fluor 549 (JF549) (*B*) or Janelia fluor 646 (JF646) (Grimm et al. 2015) (*C*,*D*). TFIIA was used at 3 nM and labeled with tetramethylrhodamine (TMR). TFIIB was used at 8 nM (*D*, *left*) or 5 nM (*D*, *right*) and labeled with Alexa 647 (*D*, *left*) or TMR (*D*, *right*). *N*_DNA_ specifies the total number of each template examined.

To better dissect how the specific hierarchy of PIC assembly is established, we used a two-color total internal reflection fluorescence (TIRF) microscope-based single-molecule imaging system (Supplemental Fig. S1; Revyakin et al. 2012) to directly visualize early PIC nucleation steps. Since TFIID (or TBP) promoter binding is generally considered the first step in PIC assembly, we began by visualizing their interaction with a TATA-box-containing promoter DNA template (Fig. 1A). As a model DNA template, we chose the synthetic supercore promoter (SCP1) with consensus TATA, Inr, and DPE elements that we showed previously can support robust transcription under our single-molecule imaging platform (Juven-Gershon et al. 2006; Revyakin et al. 2012). Because TFIID DNA binding typically covers −47 to +68 with respect to the transcription start site (+1) (Nakajima et al. 1988; Cianfrocco et al. 2013), we designed the template to span from position −63 to position +90. We reasoned that this would provide sufficient space for all of the known protein–DNA interactions within the PIC to occur under our single-molecule imaging conditions (Robert et al. 1998; Kim et al. 2000; Zhang et al. 2015). This DNA was immobilized onto the specifically functionalized imaging surface via biotin–streptavidin conjugation, with a fluorophore attached to the other end for detection (Supplemental Fig. S1B). As an internal control for binding specificity, we immobilized, within the same field of view for single-molecule imaging, another DNA template of the same length with mutations in the TATA, Inr, and DPE elements, which abolish TFIID binding and transcription (Juven-Gershon et al. 2006; Revyakin et al. 2012). After high-resolution mapping of each DNA template (Revyakin et al. 2012), we used a restriction enzyme to remove the free end of the DNA (by 9–11 nucleotides [nt]) where the fluorophores were attached. This helps prevent nonspecific interactions mediated by some fluorophores (data not shown). We then injected site-specifically dye-labeled and transcriptionally active TFIID or TBP (Supplemental Figs. S2, S3)—at concentrations relevant for transcription initiation—into the imaging chamber and monitored their interaction with the DNA templates simultaneously in real-time at an imaging rate of 2.5 Hz (Fig. 1A). Fluorescent signals from each labeled protein factor that mapped to within ∼40 nm of a preregistered DNA position were considered as “colocalized” and thus represented a binding event (Supplemental Fig. S1C; Revyakin et al. 2012). Such colocalization plots revealed a striking difference between the holo–TFIID complex and the single-subunit protein TBP: TFIID binds efficiently (∼30% of supercore templates in ∼27 min) and selectively (only ∼8% of the mutant template), while TBP binding is much less specific (∼30% on both DNA templates) (Fig. 1B) and is likely related to the upper limit of template utilization for the supercore promoter as previously reported (Juven-Gershon et al. 2006). The reduced but detectable TFIID binding to the control template might be due in part to some remaining sequences that resemble promoter elements such as the motif ten element (MTE) (Juven-Gershon et al. 2006). In any case, the ability of TFIID to discriminate between promoter DNA and the control fragment was much greater than what was observed for TBP. Stable and efficient TBP binding to extended fragments of nonspecific DNA has been reported previously and may lead to promiscuous transcription initiation (Coleman and Pugh 1995). Because PIC assembly in the cell usually occurs at nucleosome-free regions spanning several hundred base pairs of DNA, our findings suggest that TFIID is also likely more selective than TBP in binding to promoter elements in vivo.

### TFIIA binding is highly TFIID/TBP-dependent

TFIIA is generally observed to be the second factor that binds to the promoter during PIC formation (Thomas and Chiang 2006), but its role in initiation has remained somewhat enigmatic. Thus, we site-specifically labeled TFIIA without significantly compromising its biological activity (Supplemental Fig. S4A,B) and then examined its behavior at the single-molecule level. Consistent with the step-wise PIC assembly model, no significant promoter binding was detected when the labeled TFIIA was imaged alone at a frame rate of 2.5 Hz (Supplemental Fig. S4C). In contrast, we found that, in the presence of TFIID or TBP, TFIIA bound promoter DNA very efficiently, with a specificity that mirrors that of TFIID and TBP, respectively (Fig. 1C). Since TBP is known to bind and bend canonical TATA-box and mutant DNA templates to the same extent (Blair et al. 2012) and since TFIIA is thought to interact with the phosphate backbone of DNA in the TBP–TATA–TFIIA tertiary complex (Geiger et al. 1996; Tan et al. 1996), also with little to no sequence specificity, we conclude that the promoter-specific binding of TFIIA is likely to be driven entirely by its interaction with TBP or TFIID. On the other hand, the binding of TFIID or TBP was not significantly affected by the presence of TFIIA, consistent with the notion that TFIIA primarily functions subsequent to TFIID and TBP binding during PIC assembly.

TFIIA is known to interact directly with TBP independently of DNA, and this interaction is thought to enhance the dissociation of TBP dimers into monomers that bind DNA efficiently (Coleman et al. 1999; Bagby et al. 2000). However, it was not clear from bulk biochemical studies whether TBP and TFIIA arrive onto DNA sequentially or simultaneously as a preassembled complex. To resolve this question, we labeled TBP and TFIIA with two distinct fluorescent colors and compared the timing of their arrival onto DNA. Interestingly, simultaneous binding of these two molecules to DNA was not observed. Instead, TBP nearly always arrived onto DNA first, followed by TFIIA (Supplemental Fig. S4D). Therefore, the TFIIA–TBP complex is either too short-lived or not active for DNA binding. We concluded that TBP binds DNA before TFIIA rather than as a preformed TBP–TFIIA complex.

### TFIID-dependent promoter binding of TFIIB requires TFIIA

In the context of TFIID and in the absence of other factors, TBP fails to contact the TATA box (Supplemental Fig. S3B; Cianfrocco et al. 2013), presumably due to inhibition by the TAF1 subunit of TFIID, which can be alleviated by TFIIA (Bagby et al. 2000). Because a composite surface provided by both TBP and DNA is recognized by TFIIB (Tsai and Sigler 2000), we reasoned that, in a simplified system, such a function of TFIIA may be required for TFIIB binding. First, we fluorescently labeled TFIIB (Supplemental Fig. S5A–C), which retained its activity in supporting transcription or enhancing TBP–promoter DNA interactions (Zhao and Herr 2002) as determined by standard bulk assays (Supplemental Fig. S5D–F). Next, we monitored the single-molecule DNA-binding behavior of tagged TFIIB in the presence of both TFIID (or TBP) and TFIIA. We found that, at our imaging rate of 2.5 Hz, TFIIB did not bind promoter DNA when present either alone or together with TFIID (Supplemental Fig. S5G). In contrast, the addition of TFIIA to the TFIID/TFIIB mixture dramatically increased TFIIB DNA-binding efficiency from nearly zero to ∼30% (the same level as observed for TFIID) (Fig. 1D). Like TFIID, this TFIIB binding is promoter-specific. Thus, under our single-molecule imaging conditions, the binding of TFIIB to the promoter DNA is dependent on both TFIID and TFIIA. This TFIIA dependence is likely due to the inhibition of TBP–TATA binding in the context of TFIID because free TBP (when bound to the DNA template containing the supercore promoter) can efficiently recruit TFIIB independently of TFIIA (Fig. 1D). We also noticed that although TBP binds the negative control template well, this interaction does not lead to TFIIB binding (Fig. 1D, right panel). In the presence of TFIIA, TFIIB binds the control DNA well (data not shown), consistent with standard gel mobility shift assay results in which a combination of TBP, TFIIA, and TFIIB shifted both the supercore and mutant DNA with similar efficiency (Supplemental Fig. S5E,F). The promoter-specific TFIIB binding (with either TFIID and TFIIA or TBP alone) reinforces the notion that a properly promoter-engaged TBP (in a DNA sequence context that allows additional TFIIB contact) is a prerequisite for appropriate TFIIB binding. This may also explain why, when TFIID was used, even with TFIIA enabling TBP recognition of the TATA box, TFIIB binding to the control TATA-less template was less efficient than that of TFIID (Fig. 1D, left panel).

### TFIIB–promoter binding is highly transient and repetitive

**Figure 2.**
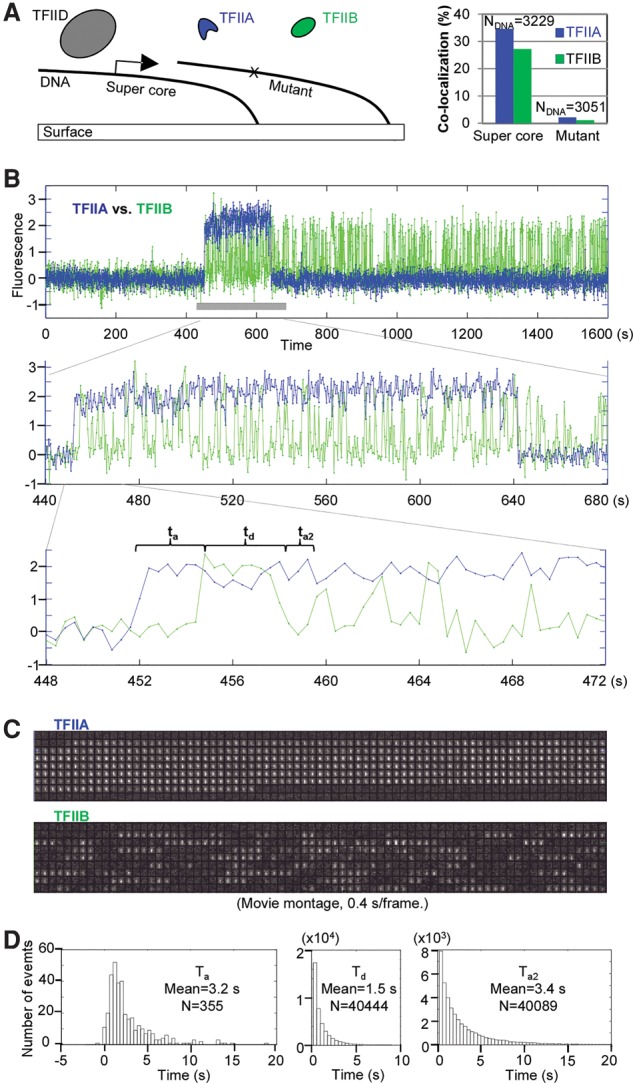
Single-molecule dynamics of TFIIB promoter binding in the presence of TFIID and TFIIA. (*A*, *left*) Scheme: Fluorescently labeled TFIIA (3 nM; TMR) and TFIIB (8 nM; Alexa 647) were mixed together with native TFIID and incubated to bind immobilized DNA templates. (*Right*) Plot of the colocalization during a 27-min (1600-sec) incubation. (*B*) A representative fluorescence time trace of TFIIA (blue) and TFIIB (green) on one DNA template containing the supercore promoter throughout the incubation time. Zoomed-in views are shown in the *middle* and *bottom* panels. Native TFIID and labeled TFIIA and TFIIB (false-colored) were used in the binding assay. The gray bar in the *top* panel demarcates the region corresponding to the movie montages shown in *C*. At the *bottom*, *t*_a_ (association time) represents the lag time between the first appearance of the TFIIA signal and the TFIIB signal, *t*_d_ (dissociation time) is the time TFIIB remains at the promoter, and *t*_a2_ (reassociation time) is the time for TFIIB to reappear at the same DNA locus. These parameters were summarized from 355 colocalized DNA molecules into histograms shown in *D* (bin size, 0.4 sec). These histograms do not fit single exponentials, suggesting that multiple steps/species might be involved in the reaction. Additionally, *t*_a_ and *t*_a2_ are likely governed by different mechanisms that are not discussed here.

The reconstitution of hierarchal and promoter-specific binding of TFIIB at the single-molecule level enabled us to take a closer look at TFIIB promoter-binding dynamics. We used native TFIID and fluorescently labeled TFIIA, whose arrival at the promoter would serve as a reference time point, together with labeled TFIIB for the single-molecule assay (Fig. 2A). As expected, both TFIIA and TFIIB showed excellent specificity in binding promoter DNA. When we examined the fluorescence time traces, we noticed that TFIIA signals lasted for minutes (Fig. 2B), which is similar to what was observed for TFIID or TBP (Supplemental Figs. S2D, S4D). We note that the observed lifetimes of TFIID/TBP/TFIIA are likely limited by photobleaching under our imaging conditions (Zhang et al. 2014), so actual residence times may be longer than the traces that we can detect. Indeed, longer residence times have been recorded under similar conditions with quantum dot-labeled TFIID that is resistant to photobleaching (Revyakin et al. 2012), and similarly longer binding times are expected for TBP (Coleman and Pugh 1995). Nevertheless, the promoter-binding interaction of TBP/TFIID/TFIIA was found to be relatively stable, as expected from the classical step-wise PIC assembly model. Surprisingly, and in stark contrast, we found the promoter binding of TFIIB (in the presence of TFIID and TFIIA) to be very transient (Fig. 2B,C). We quantified this interaction by measuring the dwell time of all TFIIB-binding events on the supercore DNA templates and found the average residence time to be 1.5 sec. We also measured how long it takes for TFIIB to bind the promoter once TFIIA is associated. We found that TFIIB has a relatively fast on rate, with an average waiting time of ∼3 sec using ∼8 nM labeled TFIIB (Fig. 2D).

To rule out fluorophore blinking as a potential source of apparent transient TFIIB promoter interaction, we mixed two TFIIB samples labeled with spectrally distinct fluorophores in one reaction and monitored their binding to the same individual DNA molecules (Supplemental Fig. S5H). We found that the two fluorescent signals appeared alternately with similarly rapid dynamics, suggesting that TFIIB indeed has a short residence time on promoter DNA, with the capability of fast repetitive rebinding. The absence of overlaps between the two signals also suggests that no more than one TFIIB molecule can occupy the promoter DNA simultaneously. Since TBP can support promoter-specific TFIIB binding in a TFIIA-independent manner, we next directly compared the dynamics of TBP/TFIIB binding to promoter DNA. In this experiment, TBP and TFIIB were each tagged with their own distinct fluorescent label to simultaneously measure binding of both factors on the same set of individual supercore promoter DNA templates. As expected, in these two-color experiments, TBP binding was found to be at least stable for minutes, while binding of TFIIB was again confirmed to be highly transient and repetitive (Supplemental Fig. S6A). The similar rapid and transient promoter-binding dynamics of TFIIB in the presence of either TBP or the TFIID complex (with TFIIA) suggests that TAF subunits of TFIID are unlikely to significantly influence TFIIB binding once TBP is released from TAF1 inhibition and engaged with the TATA-box DNA.

### Pol II is required for stable TFIIB–promoter association

**Figure 3.**
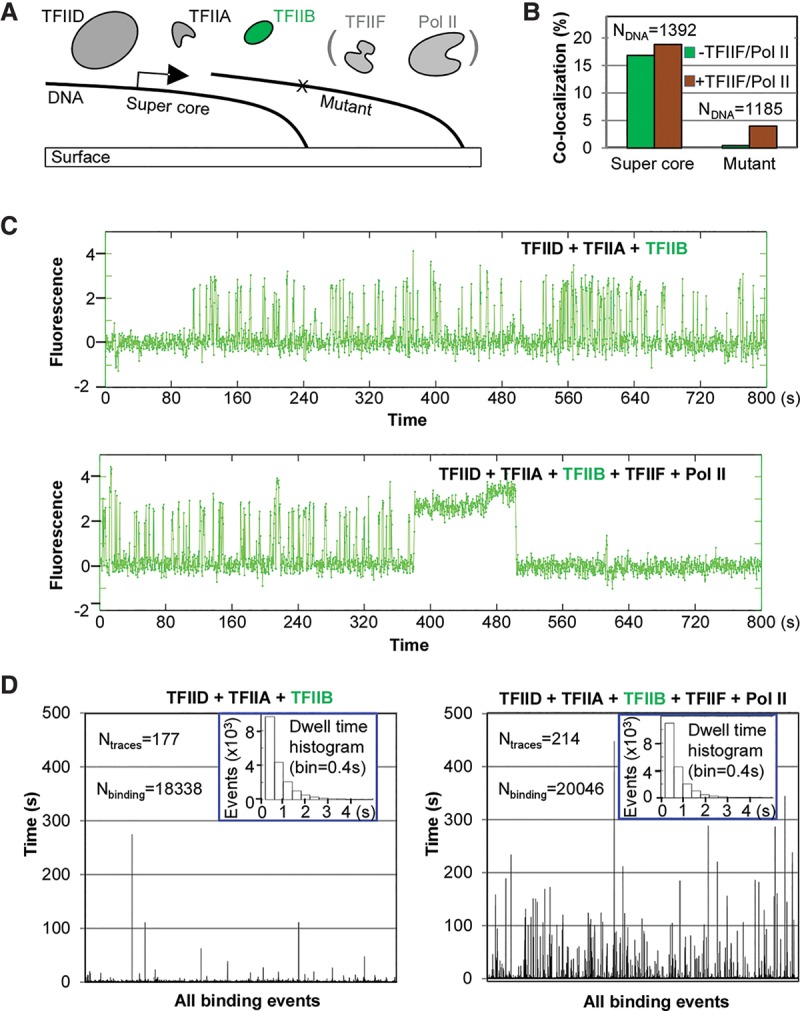
Change of TFIIB dynamics in the presence of TFIIF–Pol II. (*A*) Scheme: Fluorescently labeled TFIIB (4 nM; TMR) was mixed together with TFIID and TFIIA in the absence or presence of Pol II–TFIIF and incubated in the imaging chamber for binding to immobilized DNA templates. (*B*) Colocalization of TFIIB signals with the supercore or mutant promoter-containing DNA templates in response to the addition of TFIIF and Pol II. Results were obtained using the same set of DNA molecules incubated with two protein mixtures (first without TFIIF and Pol II and then with TFIIF and Pol II) for 13 min (800 sec). (*C*) A representative TFIIB fluorescence time trace from the same supercore DNA template in the absence (*top*) or presence (*bottom*) of TFIIF and Pol II. The transient-to-stable transition in the binding pattern occurred on approximately one-third of all DNA molecules bound by TFIIB in the presence of Pol II–TFIIF. (*D*) All TFIIB-binding events are represented by bars (height depicts residence time) to highlight the long events, which are vastly outnumbered by the short binding events in the histograms (*insets*).

The transient and dynamic TFIIB–promoter binding was unexpected based on the classical step-wise PIC formation model that envisioned each protein factor joining the growing complex by binding stably to the protein–DNA assembly (Thomas and Chiang 2006). On the other hand, indirect fluorescence recovery after photobleaching (FRAP) assays in living cells have suggested that TFIIB binding, on average, may be more dynamic (with a recovery time of seconds) than the much more stable binding of TBP (with a recovery time of minutes) (Chen et al. 2002). Therefore, the transient TFIIB promoter binding that we observed in vitro is likely to be physiologically relevant. To investigate how other components of the transcription machinery may modulate the dynamic behavior of TFIIB, we supplied TFIIF and Pol II to our reaction, which already contained TFIID, TFIIA, and TFIIB (Fig. 3A). TFIIF and Pol II often preassemble and are thought to join the promoter after TFIIB (Thomas and Chiang 2006). We found that, after adding TFIIF and Pol II, transient and repetitive TFIIB promoter binding still occurs in a promoter-specific manner (Fig. 3B,C). Indeed, the histogram of all binding events suggested that the highly transient binding mode (<1∼2 sec) is still fairly prevalent (Fig. 3D insets). However, in the presence of TFIIF and Pol II, it was striking to find a population of stable TFIIB-binding events that followed the train of transient interactions and lasted for minutes (likely limited by photobleaching) (Fig. 3C). The number of these long-binding events, although outnumbered by the repetitive transient bindings, increased dramatically in response to the addition of TFIIF and Pol II. This point became obvious when we plotted all binding events against their dwell time, which “suppressed” the transient bindings to the baseline (Fig. 3D).

The characteristic transient-to-stable transition was observed among a fairly high percentage of all traces of TFIIB binding, suggesting a process involving the initial formation of a TFIID–TFIIA–promoter complex followed by the joining of TFIIB that is stabilized by TFIIF and Pol II. To rule out the possibility that TFIIF and/or Pol II can directly deposit TFIIB in the absence of a TFIID–TFIIA subassembly, we repeated the experiments with individual factors omitted (Supplemental Fig. S7). Not surprisingly, without TFIID, no significant level of specific TFIIB binding was detected even in the presence of TFIIA, TFIIF, and Pol II (Supplemental Fig. S7A), confirming the essential role of TFIID in the assembly of a complex for stable TFIIB association. In the absence of TFIIA, promoter-specific TFIIB binding was still observed (Supplemental Fig. S7A), including significant amounts of long binding events (Supplemental Fig. S7B, top left panel). This is expected because transcription (which should require stable engagement of TFIIB) from this promoter can be TFIIA-independent (Zhang et al. 2015). Consistent with the role of TFIIA in facilitating TBP–TATA engagement as a prerequisite to repetitive and transient TFIIB binding, omitting TFIIA significantly reduced the probability of TFIIB binding (3005 events observed in 145 traces compared with 12,709 bindings in 135 traces in the presence of TFIIA) (Supplemental Fig. S7B, top left vs. bottom right panels). In the absence of TFIIF or Pol II, repetitive and transient TFIIB binding was observed, but stable association was essentially eliminated (Supplemental Fig. S7B, top right and bottom left panels), suggesting that both TFIIF and Pol II are required for stable TFIIB–promoter engagement. Therefore, the transient-to-stable transition of TFIIB binding likely reflects the step-wise assembly of the PIC that requires TFIID, TFIIA, TFIIB, TFIIF, and Pol II. When TBP was used in place of TFIID (TFIIA-independent), we observed the same transition from transient and repetitive TFIIB binding to longer interactions upon addition of TFIIF and Pol II (Supplemental Fig. S6B), suggesting that this transition to stable binding is likely intrinsic to the “core” components of the Pol II machinery: TBP and TFIIB.

**Figure 4.**
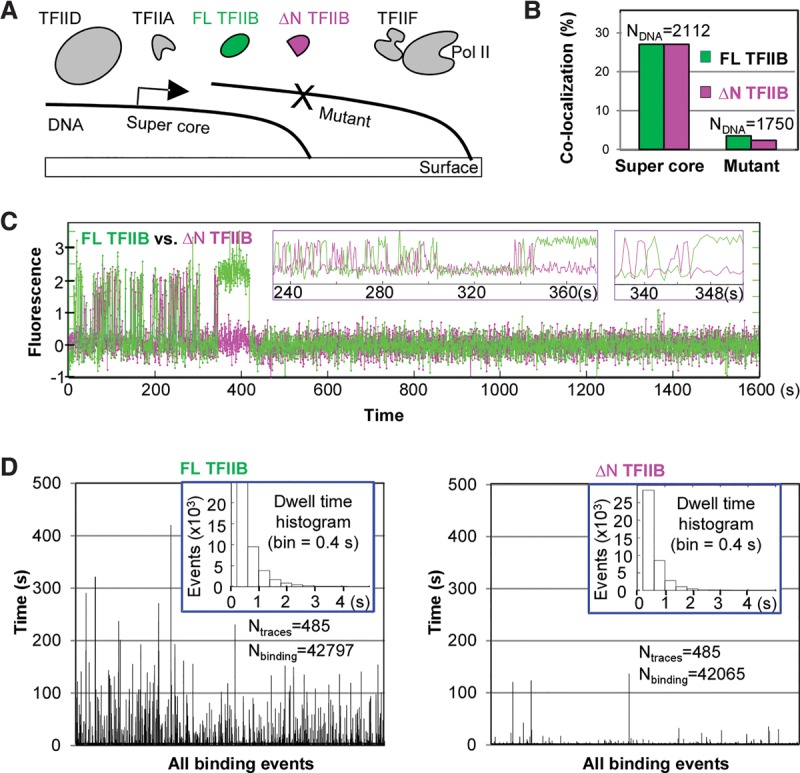
The N-terminal domain of TFIIB is required for the transient-to-stable binding transition. (*A*) Scheme: Differentially labeled FL and N-terminal (1–106)-deleted (ΔN) TFIIB (4 nM each; false-colored in green and magenta, respectively) were incubated with unlabeled (black) TFIID, TFIIA, TFIIF, and Pol II for binding to immobilized DNA templates. (*B*) Colocalization of FL and ΔN TFIIB to the supercore or mutant promoter-containing DNA templates during a 27-min (1600-sec) incubation. (*C*) A representative fluorescence time trace showing the binding of FL and ΔN TFIIB proteins to a DNA template containing the supercore promoter. *Insets* show zoomed-in views. Transient to stable TFIIB binding was observed with ∼50% of the DNA bound by TFIIB, and, among all of these transitions, most of the stable bindings (238 out of 240) were from the FL TFIIB. (*D*) All TFIIB-binding events are represented by bars (height depicts residence time) to highlight the long events, which are vastly outnumbered by the short ones in the histograms (*insets*).

Our single-molecule analysis suggests that Pol II–TFIIF can modulate the behavior of TFIIB, although TFIIB was originally thought to simply bind to the promoter and then recruit Pol II and TFIIF in the step-wise PIC assembly model. Indeed, one can postulate that this dynamic behavior of TFIIB could serve as a checkpoint for PIC formation in which assembly proceeds only when Pol II–TFIIF are available. At the same time, activators targeting TFIIB, such as VP16 (Lin et al. 1991), could potentially stabilize this transient TFIIB promoter binding and provide more time for the Pol II–TFIIF to engage, thus enabling an interesting mechanism of regulation. Consistent with our in vitro stabilization of TFIIB promoter binding by Pol II–TFIIF, genetic analysis suggested that, in live cells, stable promoter engagement of TFIIB requires proper contact with Pol II (Elsby et al. 2006). This Pol II association has been shown to be dependent primarily on the N-terminal third of TFIIB (amino acid residues 1–106) (Sainsbury et al. 2013), while the remaining TFIIB C terminus (amino acid residues 107–345) is responsible primarily for the binding to a TBP–TATA complex (Tsai and Sigler 2000). We therefore reasoned that the C terminus of TFIIB might be responsible for transient promoter-binding activity, while the Pol II-induced transition in binding dynamics might be dependent on the N terminus. To check this hypothesis, we labeled an N-terminally truncated (ΔN) TFIIB with a fluorophore that is spectrally distinct from a label used for the full-length (FL) protein and supplied both the ΔN and FL TFIIB simultaneously with TFIID, TFIIA, TFIIF, and Pol II in single-molecule-binding assays (Fig. 4A). We found that both FL and ΔN TFIIB proteins can colocalize with promoter DNA specifically (Fig. 4B). Moreover, both proteins still displayed the rapid short-lived promoter-binding events with comparable residence times (Fig. 4C,D). However, the transient-to-stable transition of TFIIB binding induced by Pol II–TFIIF was observed only for the FL TFIIB (Fig. 4C,D). These results suggest that while both ΔN and FL TFIIB can interact with promoter DNA, only FL TFIIB containing the N-terminal Pol II-interacting domain can transition to stable binding, presumably via a mechanism requiring contact with Pol II.

### N-terminal deletion reduces TFIIB residence time in vivo

**Figure 5.**
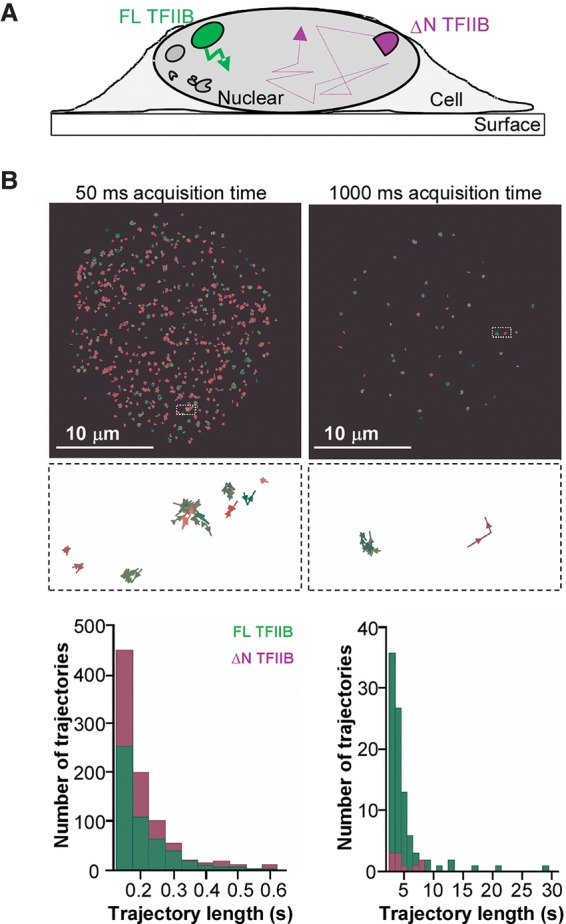
The N-terminal deletion of TFIIB increases its dynamics in live cells. (*A*) Scheme: Differentially labeled FL and ΔN TFIIB (false-colored in green and magenta, respectively) were introduced into cells by bead loading and imaged simultaneously within a living nucleus by single-molecule microscopy. At certain acquisition times, fast-moving molecules were blurred (thin dashed lines), while stably bound molecules were detected (thick solid lines). (*B*) Trajectories of FL (shades of green) and ΔN (shades of magenta) TFIIB detected by live-cell single-molecule imaging acquired with 50-msec (*left*) or 1000-msec (*right*) exposure times for a total of 100 sec. (*Top*) All TFIIB molecules detected in one nucleus for at least three consecutive frames were plotted as trajectories ([left] 539 FL and 923 ΔN TFIIB; [right] 93 FL and 11 ΔN TFIIB). (*Middle*) *Insets* (2 × 1-µm) highlighting example trajectories (color shades indicate different molecules). (*Bottom*) Histogram of all trajectories lasting three or more frames. Each trajectory was interpreted as one stable association event because the displacement was well within the diffraction limit and system variations (such as chromosome fluctuation).

Next, we set out to confirm that the unexpected, highly dynamic behavior of TFIIB that we observed in vitro also occurs in living cells using imaging modalities distinct from and complementary to classical FRAP measurements. FL and ΔN TFIIB proteins labeled with two distinct fluorophores in vitro were introduced into live U2OS cells by a “bead-loading” method (Hayashi-Takanaka et al. 2011), and their movement was tracked simultaneously using a high-sensitivity multicamera single-molecule live-cell imaging system (English and Singer 2015). We reasoned that if a molecule has moved significantly during a single-image acquisition time, its fluorescent signal will be blurred and not detectable. With longer camera exposure times, the fluorescent signal from any transcription factor that is not specifically bound to chromatin will be motion-blurred due to its fast diffusion properties, and its diffuse fluorescence signal will be overwhelmed by the cellular autofluorescence background. In contrast, chromatin polymer diffusion dynamics occurs on a much slower time scale, and hence the fluorescence of bound transcription factors will appear effectively as easily detectable immobile fluorescent particles even at longer camera exposure times (Elf et al. 2007; Xie et al. 2008). Thus, by comparing signals of the two TFIIB species at different frame rates, we would be able to compare their potentially distinct dynamics (Fig. 5A).

We were able to detect fluorescent signals from both FL and ΔN TFIIB molecules simultaneously within a nucleus of a living cell with an imaging acquisition time of 50 msec, suggesting that both species contain molecules that are “bound” for ≥50 msec (Supplemental Fig. S8A). We fitted the raw images of each frame to identify point spread functions (PSFs) corresponding to single-molecule signals and observed significantly higher standard deviations for the ΔN mutant (184.5 nm compared with 164.5 nm for FL TFIIB) (Supplemental Fig. S8B), suggesting that some of the ΔN molecules likely were mobile during the 50-msec acquisition periods. We next measured the number of frames each molecule can be traced as “trajectories” to estimate the observation time (Fig. 5B, left). We found that both TFIIB species largely remain stationary for less than half a second, which is likely a limit imposed by intrinsic photobleaching of our fluorophore. Therefore, we increased the acquisition time to 1 sec, which requires 20-fold less excitation laser power. Under these conditions, we detected very few “immobile” ΔN TFIIB molecules when compared with FL TFIIB, with the former having slightly greater standard deviations of PSFs (Supplemental Fig. S8B). More strikingly, under these conditions, the trajectories of detected ΔN TFIIB lasted less than several seconds, while those for FL TFIIB were detected up to 30 sec, suggesting that only FL TFIIB molecules exhibit a significant proportion with stable binding in live nuclei (Fig. 5B right). This observed difference in mobility between the FL and ΔN TFIIB cannot be explained by potentially different rates of photobleaching intrinsic to the two fluorescent labels because reimaging of the same samples with a shorter acquisition time of 100 msec allowed the detection of both TFIIB species at comparable levels (Supplemental Fig. S8C,D). These results suggested that the N-terminal deletion renders TFIIB molecules more dynamic in nuclei and that the behavior of TFIIB in live cells is consistent with what we observed in vitro. We postulate that ΔN TFIIB spends much more time in a rapid on/off binding mode, while a fraction of FL TFIIB can become stabilized, perhaps as a result of relatively stable binding to promoter/DNA aided by direct protein–protein interactions with Pol II via the N-terminal domain, a requisite step toward formation of a productive PIC assembly leading to transcription initiation.

## Discussion

We undertook a mechanistic analysis of the early steps in the assembly of human Pol II transcription machinery on promoters using an integrated single-molecule imaging platform. Decades of biochemical, structural, and genetic studies have identified the basic players of the Pol II system and their interactions and linked mutations to functions (Thomas and Chiang 2006). These studies have revealed a sophisticated network of coordinated molecular interactions driving gene-specific regulation of transcription (He et al. 2016). Within this network, the function of a transcription factor was often found to be context-dependent. For example, TFIIA was found to be required for only some promoters, and both stimulatory and inhibitory effects have been associated with the TFIIB response elements (BREs) of the core promoter. It has been challenging to decipher the underlying mechanisms dictating this context dependency partly because of the difficulty for traditional biochemical assays to account for variable context and reveal reversible stochastic behaviors in real time. Moreover, biochemical and genetic studies generally measure only the end products and cannot dissect transient intermediates. Our single-molecule imaging system monitors molecular interactions occurring at spatially separated individual DNA templates with high temporal resolution, thus allowing us to directly compare the behavior of individual molecules in the same reaction as well as the same collection of molecules at different steps of the reaction.

The convoluted relationships between various components of the eukaryotic transcription system became clearer when we directly compared the distinct promoter-binding properties of TBP versus TFIID and their dependence on TFIIA in recruiting TFIIB. Within the Pol II machinery, TBP and TFIIB are the two polypeptides most conserved between eukaryotes and archaea (Kosa et al. 1997). In fact, under specific in vitro conditions, TBP and TFIIB are sufficient to support promoter-dependent transcription by Pol II (Parvin and Sharp 1993). TBP binding and bending of the TATA (or TATA-like) DNA sequence is essential for promoter binding of TFIIB (Kosa et al. 1997), which in turn recruits and triggers Pol II activity (Sainsbury et al. 2013). Therefore, controlling the promoter delivery of TBP appears to be a key step for initiating transcription.

Nonspecific TBP–DNA interactions have been well documented and are known to drive promiscuous transcription initiation from long (>100-bp) DNA templates (Coleman and Pugh 1995). Under similar assay conditions using 153-bp DNA fragments, we found TFIID promoter binding to be much more sequence-specific than TBP. This is consistent with two known aspects of TAF function within the TFIID complex: (1) The N terminus of TAF1 can bind and inhibit the DNA-binding surface of TBP (Liu et al. 1998; Bagby et al. 2000), which prevents nonspecific DNA binding. (2) Multiple TAF subunits (TAF1, TAF2, TAF6, TAF9, etc.) can recognize additional core promoter elements (Juven-Gershon and Kadonaga 2010; Louder et al. 2016), which increases the specificity. Consistent with the inhibition of TBP–TATA DNA interactions within the TFIID complex, we observed that the promoter-bound TFIID is incapable of recruiting TFIIB in the absence of TFIIA. In contrast, TBP can recruit TFIIB even without the aid of TFIIA. This is the first direct evidence for a functional consequence of TBP inhibition in the context of TFIID at the promoter. Importantly, we also found that addition of TFIIA, which is thought to relieve TBP inhibition by TAF1 (Bagby et al. 2000; Cianfrocco et al. 2013), enabled TFIID to recruit TFIIB efficiently. These findings suggest that by packaging TBP into the TFIID complex, eukaryotes require the services of an additional regulatory factor, TFIIA, to unlock the latent TATA–DNA-binding capacity of TBP. It has been reported that TFIIA is dispensable at some promoters for transcription initiation activity in vitro, suggesting that alternative mechanisms and factors may exist to overcome this TBP inhibition by TAF1, adding further points of control through regulation. This more elaborate network of checks and balances also affords higher eukaryotes the advantage of diversifying the repertoire of selective protein–protein and protein–DNA interactions enabled by the TAFs serving as targets of an expanded universe of activators and modulators of transcription. The evolution of these additional components of the PIC to regulate the delivery and function of TBP also expanded the range of functional core promoter elements to include a large class of apparently TATA-less promoters capable of being targeted by TFIID. Importantly, both the TFIID complex and TFIIA are major targets within the Pol II machinery, interacting with activators (Thomas and Chiang 2006) while TFIID also recognizes different control signals from the chromatin (Jacobson et al. 2000; Lauberth et al. 2013). Taken together, these findings suggest that TFIID and TFIIA constitute an efficient platform to integrate multiple regulatory signals controlling TBP–promoter binding and transcription initiation.

Our work revealed an unexpected dynamic behavior of TFIIB interactions with the promoter, adding a new twist to the generally accepted step-wise model of PIC assembly that envisioned GTFs arriving at the promoter one after another, binding stably, and awaiting the arrival of the next factor. The hierarchy of TFIID, TFIIA, and TFIIB in promoter binding; the on rates of TFIID and TBP; and the minutes-long residence time of TFIID, TBP, and TFIIA that we recorded here are all consistent with previous reports (Coleman and Pugh 1997; Revyakin et al. 2012) and the conventional assembly model. However, the promoter binding of TFIIB turned out to be a surprise, as it exhibited much more rapid transient binding and rebinding on the order of a few seconds. Importantly, this rapid on/off behavior becomes stabilized only after the arrival of Pol II–TFIIF, suggesting an important “checkpoint” function of Pol II–TFIIF in controlling TFIIB promoter binding. This dynamic behavior and its transition to stable interaction had escaped classical biochemical methods, resulting in the simplified step-wise deterministic assembly model. We envision that the stabilization of TFIIB could be achieved by two not mutually exclusive mechanisms: (1) Pol II–TFIIF joins the PIC during transient TFIIB binding and locks the ternary complex into position by engaging more protein–protein and protein–DNA interactions, and/or (2) a fraction of TFIIB preassociates with Pol II–TFIIF prior to entry into the TFIID–TFIIA–DNA partial assembly as a preassembled partial complex. We postulate that this stabilization process could present an advantageous mechanism during PIC formation to exercise a potentially important point of control. For example, factors stabilizing TFIIB binding may increase the chance for this transient stage to be captured by Pol II and TFIIF. Alternatively, factors modulating direct Pol II–TFIIB interactions may regulate the abundance of this presumptive preassembled form, which in turn may modulate PIC assembly.

Comparing the step-wise pathway versus using partially preassembled subcomplexes during PIC formation, our data generally favor the former and emphasize the integration of multilevel regulatory signals via different components of the transcription machinery. However, these two models are not necessarily mutually exclusive. We also caution that our preferred model is based on data from one synthetic promoter containing multiple canonical core promoter elements, whereas in vivo PIC assembly could use diverse pathways, depending on the composition of promoter structures and the local chromatin environment. Using a complementary single-molecule in vitro assay system, Goodrich and colleagues (Horn et al. 2016) reported recently that TBP–TFIIB–TFIIF–Pol II can form a stable quadruple complex capable of supporting transcription, consistent with a partial preassembly model. In the future, simultaneous monitoring of multiple protein factors binding to immobilized promoter DNA, particularly when coupled to real-time nascent RNA detection (Zhang et al. 2014), should help clarify how prevalent such hypothetical preassembled complexes may be and their functional relevance for promoter-specific gene regulation.

In a curious exception, contrary to the essential role of TFIIB and TAFs in directing canonical Pol II promoter transcription, TFIIB and TAFs appeared to be dispensable for transcription of the core histone genes in *Drosophila* (Guglielmi et al. 2013). It was postulated that perhaps a related but unidentified paralog of TFIIB may substitute for TFIIB under this specialized gene transcription system for the highly reiterated *Drosophila* histone genes. The utilization of alternative core components and diversified composition of the PIC is a plausible evolutionary strategy to expand transcription initiation mechanisms to accommodate a greater repertoire of gene-specific regulation required by the physiological and developmental needs of higher eukaryotes.

Our TFIIB dynamic measurements help resolve an apparent controversy between the expectations of the classical PIC assembly model (stable TFIIB binding “recruits” Pol II) and fast dynamics (binding half-life of a few seconds) of TFIIB observed in vivo (Chen et al. 2002). Interestingly, our direct in vivo single-molecule imaging studies tracking the same fluorescently labeled TFIIB proteins used in our in vitro assays also revealed fast dynamics in live nuclei. In vivo, observation time is limited by photobleaching under our experimental conditions. Additionally, in the presence of TFIIE, TFIIH, and nucleoside triphosphate substrates in live nuclei, Pol II may escape the promoter much more efficiently than observed under our in vitro reconstitution conditions. This may increase the apparent dynamics of TFIIB, which is known to be released during promoter clearance when the nascent RNA reaches ∼12 nt in length (Cabart et al. 2011). However, despite these caveats, we observed a population of FL TFIIB in vivo with relatively longer binding time up to tens of seconds that likely corresponds to the stable TFIIB promoter-binding events observed in our in vitro assays. Most importantly, our in vivo measurements confirmed that deletion of the N-terminal Pol II-interacting domain of TFIIB results in even shorter residence times of TFIIB in live cells, consistent with the potential role of the TFIIB–Pol II interaction as a checkpoint for inducing stable TFIIB promoter binding.

In summary, our single-molecule studies have uncovered aspects of the assembly process that had eluded detection previously: a highly dynamic and rapidly reversible binding of TFIIB during the formation of the PIC. These observed rapid dynamics of TFIIB binding provide an opportunity for a host of other cellular factors to regulate PIC assembly. We speculate that such dynamic interactions may play key roles during gene regulation and that other rapid and transient modalities of binding by key transcription factors remain to be discovered using approaches such as single-molecule imaging platforms.

## Materials and methods

### Protein purification and labeling

Transcription factors, unless otherwise specified, were purified as described previously (Revyakin et al. 2012; Zhang et al. 2015). Recombinant proteins were expressed in *Escherichia coli* and purified according to manufacturer-suggested methods. GST fusion proteins were purified by glutathione-sepharose 4B resin (GE Healthcare), and (His)_6_-Halo fusion proteins were purified by Ni-NTA agarose resin (Qiagen) to >90% purity as determined by SDS-PAGE Coomassie Brilliant Blue-stained gels. Details of protein labeling are in the Supplemental Material.

### In vitro transcription biochemistry and DNA templates

In vitro transcription using plasmid DNA template containing the supercore promoter (SCP1) (Juven-Gershon et al. 2006) was as described previously (Revyakin et al. 2012). The sequence of the supercore promoter-containing DNA template used for single-molecule-binding assays was the PCR-amplified fragment CATAAC*CATATG*TATCATACACATACGGTACT**TATATAAG** GGGGTGGGGGCGCGTTCGTCC**TCAGTCG**CGATCGAACA CTCGAGCCGAGCAG**ACGTGCC**TACGGACCGCAAGCTTC CCTATCCCTTATCTTAACCACTCCAATTACATACACC. The regions corresponding to the primers used for PCR amplification are underscored, and the key core promoter elements are in bold (from 5′ to 3′: TATA box, Inr, and DPE, with the transcription start site inside of the Inr also underscored). The upstream primer was labeled with Cy3 or Atto633 at the 5′ end (Integrated DNA Technology) and also contains a site recognized by the restriction enzyme Nde I (CATATG in italic), allowing the removal of the dye after initial mapping of the DNA molecules. The downstream primer has a biotin tag at the 5′ end, allowing for surface immobilization.

The control DNA template sequence with mutations in the TATA box, Inr, and DPE (mutated residues are in lowercase) was as follows: CATAAC*CATATG*TATCATACACATACGGTAC**acgTatgt**GGGGGTGGGGGCGCGTTCGTCC**TgtGaCa**CGATCGAACACTCGAGCCGAGCAG**cataGCC**TACGGACCGCAAGCTTCCCTATCCCTTATCTTAACCACTCCAATTACATACACC.

The primer extension assay to check Pol II transcription from DNA templates immobilized on the spin-coated imaging surface (see below) was carried out with Atto633-labeled supercore DNA template following previously described procedures (Revyakin et al. 2012).

Gel mobility shift assays were carried out in a buffer containing 5% glycerol, 12.5 mM HEPES (pH 7.9), 6 mM MgCl_2_, 50 mM KCl, 50 µM EDTA, 40 ng/µL yeast tRNA, 2 ng/µL poly(dG:dC), 50 µg/mL bovine serum albumin, 0.1% Tween 20, and 0.005% NP40. The DNA templates as described above were used at 10 nM final concentration (total volume of 20 µL) and incubated with 20 or 60 ng of TBP, TFIIA, and TFIIB (alone or in combinations) for 1 h at 4°C followed by 10 min at 23°C. The protein–DNA complexes were separated by electrophoresis in 0.8% agarose gel (10 v/cm 1× TBE) for 30 min at 4°C and imaged by Typhoon Trio^+^ scanner (GE Healthcare).

### In vitro single-molecule imaging and data analysis

The TIRF microscope instrumentation and imaging chamber preparation were as described previously with minor modifications (Revyakin et al. 2012; Zhang et al. 2014).

Imaging surface passivation was achieved by spin coating. First, acidic piranha-treated coverslides (VWR, 48393280) were first spin-coated by MCC Primer 80/20 (MicroChem, P021020) at 500 rpm for 10 sec followed by a spin at 3000 rpm for 20 sec. The slides were then cured for 2 min at 100°C before a second spin coating at 2000 rpm for 20 sec with a solution in toluene with the following: 0.5% polystyrene (Sigma, 182427), 0.005% azide-terminated polystyrene (Sigma, 699772), and M280 streptavidin beads (ThermoFisher, 11205D) (2 µL of beads for 200 µL of polystyrene solution prerinsed with methanol). After assembling into an imaging chamber with the spin-coated sides facing inward, biotin was conjugated by click chemistry as reported (Presolski et al. 2011) with the following key specific reagents: biotin-PEG5000-C12-Alkyne (Baseclick) and THPTA-ligand (Baseclick). The chambers were rinsed with standard PBS buffer supplemented with 0.1% Tween 20 before use. In single-molecule protein-binding experiments, the fluorophores attached to the free end of DNA were usually removed by restriction digestion (an NdeI digestion site was introduced by the primer used for PCR amplification, and the removal was ∼95% efficient) after DNA mapping. This procedure was developed because we noticed that Atto633 fluorophore nonspecifically interacts with Atto565-Tris-NTA fluorescent signals in our TFIID-binding experiments.

All protein mixtures were made with a buffer containing 4.5% glycerol, 11.3 mM HEPES (pH 7.9), 5.6 mM MgCl_2_ , 45 mM KCl, 45 µM EDTA, 3.6 ng/µL yeast tRNA (Sigma) (additionally purified with protease K treatment and phenol/chloroform extraction), 0.1 ng/µL poly(dG:dC), 0.9 mM Trolox (Sigma), 2.3 mM protocatechuic acid (Sigma), 0.09% Tween 20 (EMD Chemicals), and 0.0045% NP40 (EMD Chemicals). Protocatechuate dehydrogenase (10 µg/mL; raw material purchased from Toyobo and further purified in-house to remove contaminating nucleases activity) (Zhang et al. 2014) was added to all mixtures immediately before injection into the imaging chamber.

The opening of the imaging chamber was bathed with infusing pure nitrogen gas to prevent contact with oxygen in the air. All single-molecule imaging movies were taken at 2.5 Hz. Colocalization analysis and generation of single-molecule fluorescence time traces were essentially as reported previously (Revyakin et al. 2012; Zhang et al. 2014) and are also described in the legend for Supplemental Figure S1. Kinetic analysis for binding and dissociation times of labeled molecules from single-molecule traces was done using MatLab scripts developed previously (Tsai et al. 2012). More specifically, the fluorescent signal from a labeled protein factor was measured within a 5 × 5-pixel (1 × 1-µm) region of interest centered at the bound DNA molecule (identified by statistical colocalization analysis) (see the legend for Supplemental Fig. S1). The on or off state of binding was determined by an empirical intensity threshold, which allowed the registration of the time of association and dissociation. Histograms were plotted, and means of dwell time and waiting time were computed in MatLab using built-in functions.

### In vivo single-molecule imaging and data analysis

U2OS cells were grown in DMEM (Thermo Scientific) supplemented with 10% FBS. Prior to experiments, cells were plated on precleaned 25-mm diameter coverslips (Electron Microscopy Sciences) in standard six-well culture plates and bead-loaded with fluorescently labeled FL and ΔN(1–106) mutant TFIIB at concentrations of ∼0.3 µM. After removing DMEM, 5 µL of solution was pipetted onto the cells, and ∼100-µm glass beads (Sigma Aldrich) were distributed over the cells. The six-well culture plate was then tapped five times, and DMEM was added back to the cells. Three hours after bead loading, the cells were washed three times with phenol-red-free complete DMEM to remove glass beads, and the coverslip was mounted into a metal cell chamber (Life Technologies) in preparation for imaging. The bead-loading procedure has been described in detail (Hayashi-Takanaka et al. 2011).

During imaging, cells were maintained at 37°C, 5% CO_2_, and constant humidity using a Tokai-hit stage-top incubator (INUP-PPZI-F1). Simultaneous two-color live-cell single-molecule imaging and tracking experiments were recorded on a custom-built three-camera RAMM frame (ASI) microscope using an 1.4-NA PLAPON 60× OSC objective (Olympus) and a 300-mm focal length tube lens (LAO-300.0, Melles Griot), resulting in 100× overall magnification. The microscope has been described in detail in English and Singer (2015). An acquisition board (National Instruments) controlled a 555-nm CrystaLaser as well as a 637-nm laser from a Stradus 637-140 (Vortran). Power levels for both 555-nm as well as 637-nm laser illumination were estimated at 1 kW/cm^2^ at the sample for imaging at 20 fps, 500 W/cm^2^ for imaging at 10 fps, and 50 W/cm^2^ for imaging at 1 fps.

The Localizer image analysis package (Dedecker et al. 2012) was used to track single particles in acquired live-cell movies. The following settings were chosen for particle detection and track linking: two-pixel maximum jump distance, three-frame minimum track length, eight-way adjacent tracking, 1.3 SD, and 20 GLRT sensitivity. Resulting tracks were then exported as text files using code written in Igor 6.3.4 (WaveMetrics), which was also used to make histograms of the particle trajectory lengths. Integrated fluorescence intensities were calculated and converted to photon counts using analysis routines written in Igor Pro. Localization errors were calculated using Equation 6 in Mortensen et al. (2010). Superresolution images were rendered using the software package VISP (El Beheiry and Dahan 2013).

## Supplementary Material

Supplemental Material
