# Supplementary material for: Rapid dynamics of general transcription factor TFIIB binding during preinitiation complex assembly revealed by single-molecule analysis
Source: Genes Dev. 2016 Sep 15;30(18):2106–18. doi: 10.1101/gad.285395.116 (PMC5066616; doi:10.1101/gad.285395.116)
Supplement: Supplemental Material [file supp_30_18_2106__index.html]

Supplemental Material 

# Rapid dynamics of general transcription factor TFIIB binding during preinitiation complex assembly revealed by single-molecule analysis

## Supplemental Material

**Files in this Data Supplement:**

- Supplemental\_Material.pdf
