# Supplementary material for: Rapid dynamics of general transcription factor TFIIB binding during preinitiation complex assembly revealed by single-molecule analysis
Source: Genes Dev. 2016 Sep 15;30(18):2106–18. doi: 10.1101/gad.285395.116 (PMC5066616; doi:10.1101/gad.285395.116)
Supplement: Supplemental Material [file supp_30.18.2106_Supplemental_Material.pdf]

Supplemental files for Zhang et al.

Supplemental Figure Legends

Supplemental figure S1: Experimental system and *in vitro* single-molecule co-localization algorithm. (**A**) Scheme: DNA templates were immobilized on the single-molecule imaging surface. Arrow head depicts the transcription start site. Transcription factors were incubated and the DNA binding of the fluorescently labeled molecules were monitored in real-time. (**B**) Left: Scheme of surface passivation and surface functionalization. Clean borosilicate glass substrates were spin-coated with a polystyrene layer containing azide-terminated polystyrene, to which biotin groups were attached via C12 and PEG5000 linkers, using copper-dependent click chemistry (Presolski et al., 2011). This permitted the immobilization of fluorescently labeled DNA molecules via biotin-streptavidin interactions. Surface passivation was achieved by carrying out all biochemical reactions in the presence of 0.05%~0.1% Tween 20. Right: Result of a transcription assay comparing promoter-specific transcription initiation activity by our reconstituted human Pol II system from surface immobilized DNA under our single-molecule imaging conditions (“on surface”) and freely diffusing DNA under conventional bulk biochemistry conditions (“in solution”). (**C**) Algorithm of statistical co-localization analysis to identify single-molecule protein-DNA interactions with TBP-super core promoter DNA interaction used as an example: (Step 1) Localization of DNA molecules. DNA spots in every frame (raw image on the left, 4x4 μm region of interest

(ROI), false-colored in green) of the DNA mapping movie were identified, each fitted to a 2D Gaussian pixel intensity distribution (Huang et al., 2010), to produce a dataset of *(x, y)* points (green crosses) from cumulative frames. Clusters of *(x,y)* points were identified, and the average *(x, y)* of each cluster determines the location of the respective DNA (black downward triangles) (middle and right). 6 DNA molecules are shown out of a total of 1230. (Step 2) Localization of TBP-surface interactions. TBP spots in every movie frame (representative ROI on left) were identified, localized in (x,y), and clustered as described for DNA, giving the *(x,y)* locations of TBP-surface interactions (downward pointing triangles). Only clusters containing >5 *(x,y)* points were kept for co-localization analysis. In this representative dataset, 11 TBP-surface interactions were identified in the 4000-frame movie (1600 s) and localized. (Step 3) Calculation of DNA-TBP displacements. Left is a merged color image of the ROI generated from the false-colored DNA and TBP images in Steps (1) and (2). Middle are locations of DNA molecules and TBP-surface interactions during the entire movie. Right shows the location of DNA 1, and the location of the most proximal identified TBP-surface interaction for DNA 1. The displacement (Δx, Δy) was recorded for each DNA. (Step 4) Statistical identification of DNA-TBP interactions. Left: DNA-TBP co-localization plot (Δx Δy) for the 6 representative DNA. In this plot, DNA molecules that localize within 40 nm from the origin (0,0) are statistically most likely to have experienced a TBP interaction during the entire movie. Right: DNA-TBP co-localization plot for all 1230 DNA (Δx Δy), showing a non-random enrichment within ~40 nm from the origin (0,0), indicating that a significant fraction of DNA molecules (35 % in this case, used in Figure 1B, right panel) interacted

with an identifiable TBP molecule during the 4000-frame movie, to within the statistical error determined by the localization uncertainty of 20-40 nm (Revyakin et al., 2012).

Supplemental figure S2: Fluorescent labeling of TFIID via a Tris-Ni-NTA fluorophore conjugate. (**A**) Structure of Atto565-conjugated tris-nitrilotriacetic acid compound coordinated with Ni^{2+} (Atto565-Tris-NTA) (top, X depicts ligands coordinating the Ni^{2+} cation) and the proposed binding target sequence within TFIID (bottom, amino acid residues 1146-1171 of human TAF2, with the histidine residues in red as potential Ni^{2+} ligands). (**B**) The Atto565-Tris-NTA compound recognizes a band of human TFIID separated by SDS-PAGE, corresponding to TAF2 by molecular weight. Left is a gel image with TFIID subunits detected by silver stainning. Right is the same sample stained by 3 nM Atto565-Tris-NTA and scanned using 532 nm excitation. A total of ~200 ng TFIID was loaded in each gel. Arrow head points to the position of TAF2. (**C**) Atto565-Tris-NTA, used at a concentration of 1 µM, does not affect TFIID-directed transcription. Shown is the transcription product detected by primer extension. (**D**) A representative single-molecule fluorescence time trace of a 5 x 5 pixel (~1 x 1 µm) area of imaging surface containing a single super core DNA template, obtained in the presence of ~1 nM TFIID and 5 nM Atto565-Tris-NTA (top) and its movie montage (bottom) indicating the DNA binding of the fluorescently labeled TFIID. The fluctuations in signal are likely due to the intrinsically disordered (structurally dynamic) nature of the targeted region within TAF2 (Zhang et al., 2015) and/or the structural flexibility of the compound itself.

Supplemental figure S3: Fluorescent labeling of TBP via the HaloTag. (**A**) Gel images of JF549 and JF646-labeled Halo-TBP proteins stained by Coomassie (left) or scanned using 532 nm excitation (middle, for JF549 fluorescence) and 633 nm excitation (right, for JF646 fluorescence). Arrow depicts position of free dyes. (**B**) Transcriptional activity of the HaloTag TBP fusion before (unlabeled) and after (labeled) labeling with JF549. Specified amount of TBP protein was used together with other GTFs to reconstitute transcription, and the RNA product was detected by primer extension. For the tagged TBP (molecule weight of 73 kD), 3 ng in 27.5 µl (our standard transcription reaction volume) leads to 1.5 nM final concentration. (**C**) Chemical structure of the two fluorophore-conjugated Halo ligands used for labeling.

Supplemental figure S4: Fluorescent labeling of TFIIA via the SortaseTag. (**A**) Gel images of TMR-labeled TFIIA stained by *Coomassie* (left) or scanned using 532 nm excitation (right, for TMR fluorescence). Arrows depict the positions of TFIIA α-β fusion and γ subunits. The γ was also labeled due to a glycine residue (introduced as a cloning linker at position 2) exposure after bacterial removal of the first methionine residue, which served as a label acceptor. (**B**) DNase I footprint assay monitoring the biochemical activity of TFIIA. The gel images of DNA (1 nM) digested by DNase I are shown. Highly purified TFIID alone (at ~ 1nM final concentration) failed to protect the TATA box, due to the auto inhibition of its TBP subunit by TAF1. TFIIA can overcome this inhibition. 1 nM DNA was used in all reactions. (**C**) Efficiency of co-localization

between TFIIA alone (TMR labeled, 3 nM) and DNA templates under single-molecule binding conditions. (**D**) Left is a representative single-molecule fluorescence time trace monitoring simultaneously the binding of both TBP (JF646 labeled, 2 nM) and TFIIA (TMR labeled, 3 nM) (top) and the corresponding movie montages (bottom). The disappearance of fluorescent signal most likely reflects bleaching of the fluorophore, instead of dissociation of the labeled protein. A TFIIA molecule with only one bleaching step (presumably with only one fluorophore label) was selected for simplicity. Grey bar depicts the period covered by the movie montages below. Right is the histogram of the time delay between TBP binding and TFIIA binding.

Supplemental figure S5: Fluorescent labeling of TFIIB via the unnatural amino acid strategy and the SortaseTag. (**A-C**) Gel images Alexa647 labeled TFIIB via a para-azidyl phenylalanine (pAzF) (**A**), TMR and JF646-labeled TFIIB (**B**) or its N-terminal deletion (Δ106) (**C**) via the SortaseTag. Coomassie staining and fluorescence scanning at 532 nm (for TMR) or 633 nm (for Alexa647 and JF646) are shown. “No label” contains samples of the same protein construct before labeling. (**D**) Transcriptional controls testing the biochemical activity of the labeled proteins. The N-terminal deletion (TMR-ΔN and JF646-ΔN) are expected to be inactive in this assay. For the tagged TFIIB (molecule weight of 37 kD for the unnatural-amino acid tagged, and 35 kD for the sortase tagged), 5 ng in 27.5 μl (our standard transcription reaction volume) leads to 5 nM final concentration. (**E-F**) Gel mobility shift assay monitoring DNA binding activity of Alexa 647 (**E**) or TMR (**F**) labeled TFIIB, with Cy3 or Atto633 labeled DNA templates,

respectively. TBP and TFIIA were used as indicated. Gels were scanned at 532 nm and 633 nm and presented in both channels. Arrow heads depicts positions of free probes and the possible protein-DNA complexes. (**G**) Co-localization of full-length TFIIB alone (left), or together with TFIID (right), with DNA templates under single-molecule binding conditions. (**H**) A representative single-molecule fluorescence time trace monitoring the promoter binding of TFIIB molecules labeled using two distinct fluorescent labels (4 nM each) (Alexa647, red; TMR, green) in the presence of pre-bound TBP.

Supplemental figure S6: Transient-to-stable transition of TFIIB promoter binding was also observed when TBP was used in place of TFIID. (**A**) Representative fluorescence time traces of labeled TBP and TFIIB (used together, without any other factor) binding to a super core promoter DNA is shown on the top. Bottom is a plot of ~1000 binding events with their corresponding TFIIB binding time indicated as bars along y-axis. (**B**) is the same as (**A**) except for that TFIIF and Pol II were also included in the reaction. In this case, about 1/3 of the traces showed a standard transient-to-stable transition in the TFIIB signal.

Supplemental figure S7: Single-molecule TFIIB-promoter binding controls with individual factors omitted. (**A**) Summary of co-localization of TMR-labeled TFIIB with super core or mutant DNA templates at the presence of other factors as specified. (**B**) Comparison of TFIIB binding time with different combination of GTFs as specified. Major figures have all TFIIB binding events from selected super core DNA template

represented by bars (height depicts binding time) to highlight the long events. Inserts are histograms of binding time of all events from the same set DNA molecules which are overwhelmed by the transient events.

Supplemental figure S8: Live-cell single-molecule images and controls for TFIIB dynamics. (**A**) Maximum projection of raw fluorescence intensity images of five consecutive frames of the nucleus of a living cell, at both 50 ms (left column) and 1 s (right column) acquisition times, recorded simultaneously for both FL TFIIB (top row) and ΔN TFIIB (bottom row). All four images are 25 x 25 µm in size. (**B**) Histograms of the fitted standard deviation of the point spread function for both FL (green) and ΔN (magenta) TFIIB obtained at an acquisition time of 50 ms (left) or 1 s (right). (**C**) Processed super-resolution images of FL (green) and ΔN (magenta) TFIIB obtained at three different acquisition times as specified (movie was taken in the order of from left to right), each for a total time of 100 s. All detected localizations are displayed according to their localization full-width at half-maximum, with their total numbers (n) specified. The mean and median localization errors were all between 30~40 nm. (**D**) Trajectories detected at 100 ms acquisition time (254 FL and 158 ΔN TFIIB). See Figure 5B legend for more details.

Supplemental Figures

Zhang_Fig S1

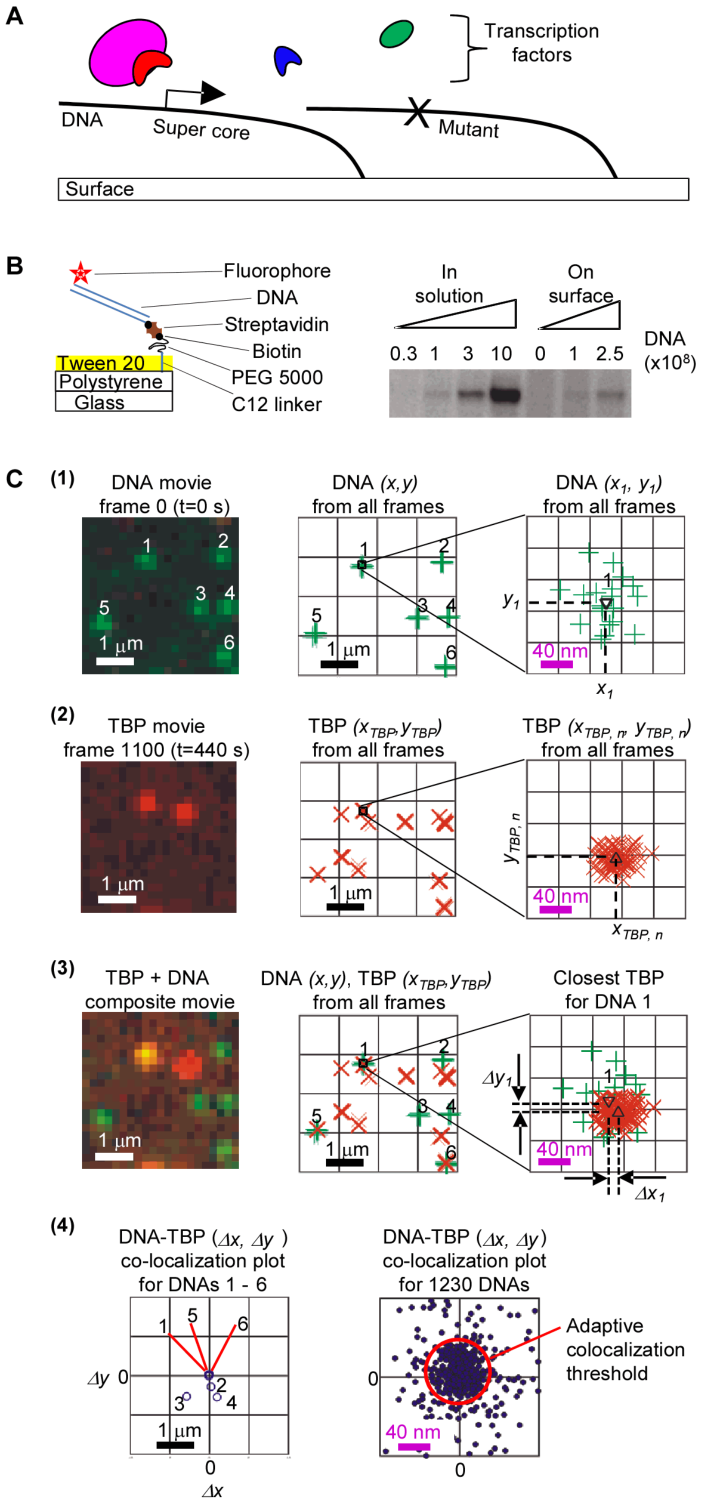

Zhang_Fig S2

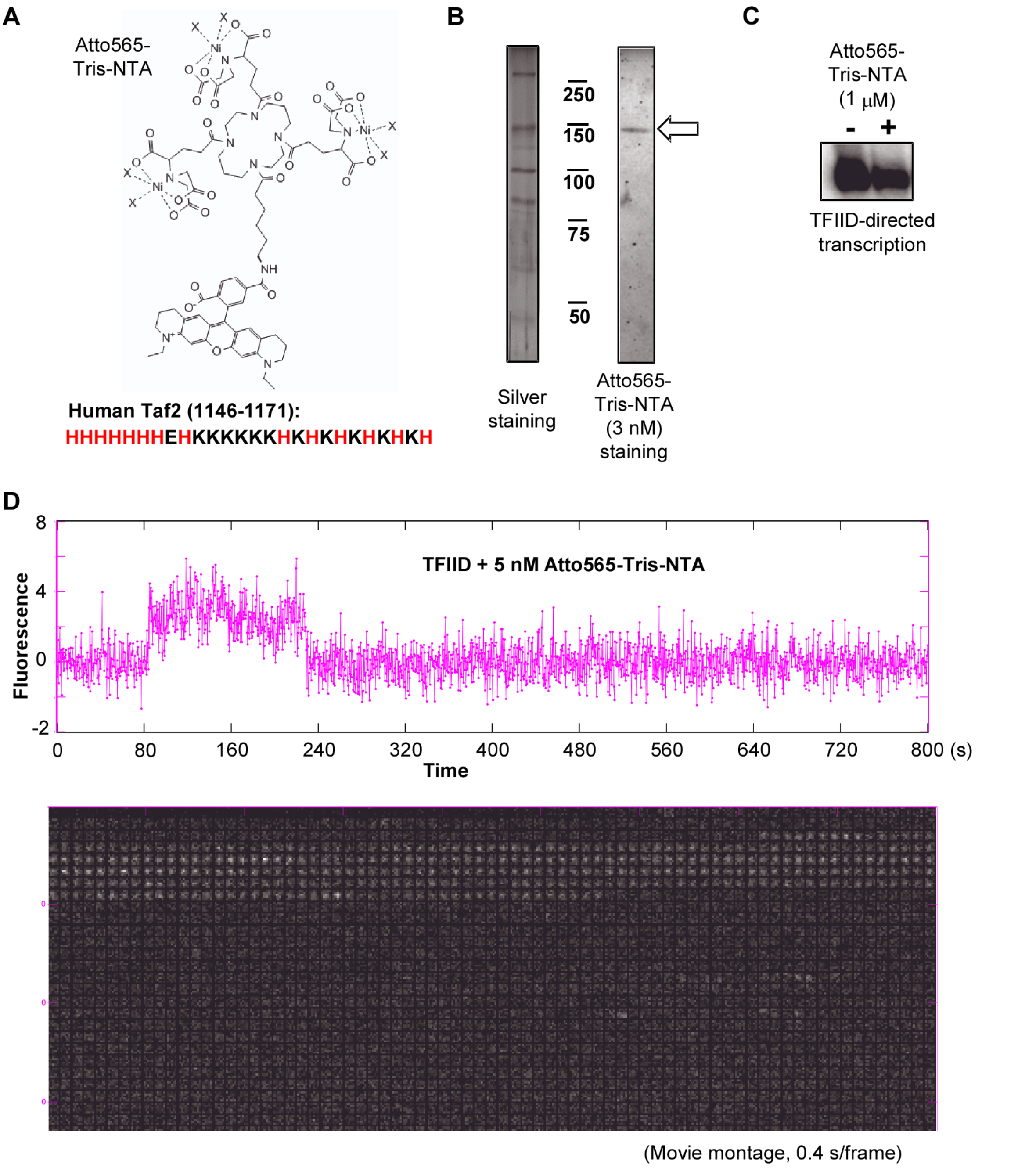
A
Atto565-Tris-NTA
Human Taf2 (1146-1171):
HHHHHHHEHKKKKKKHKHKHKHKHKH
B
250
150
100
75
50
Silver staining
Atto565-Tris-NTA (3 nM) staining
C
Atto565-Tris-NTA (1 µM)
- +
TFIID-directed transcription
D
TFIID + 5 nM Atto565-Tris-NTA
Fluorescence
Time
0 80 160 240 320 400 480 560 640 720 800 (s)
8 4 0 -2
(Movie montage, 0.4 s/frame)

Zhang_Fig S3

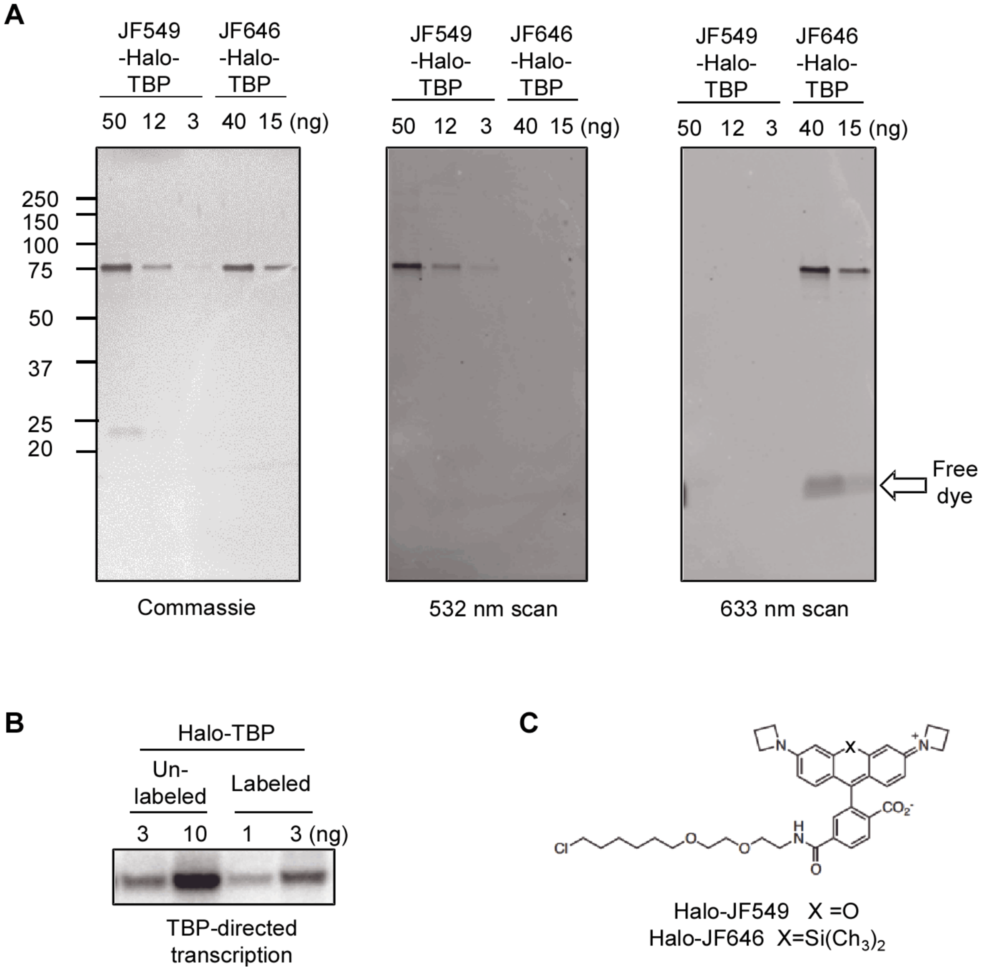
A
JF549 -Halo- TBP
JF646 -Halo- TBP
50 12 3 40 15 (ng)
250
150
100
75
50
37
25
20
Commassie
532 nm scan
633 nm scan
Free dye
B
Halo-TBP
Un- labeled
Labeled
3 10 1 3 (ng)
TBP-directed transcription
C
Cl
O
O
H N
O
N
X
N
+
CO2-
Halo-JF549 X =O
Halo-JF646 X=Si(Ch3)2

Zhang_Fig S4

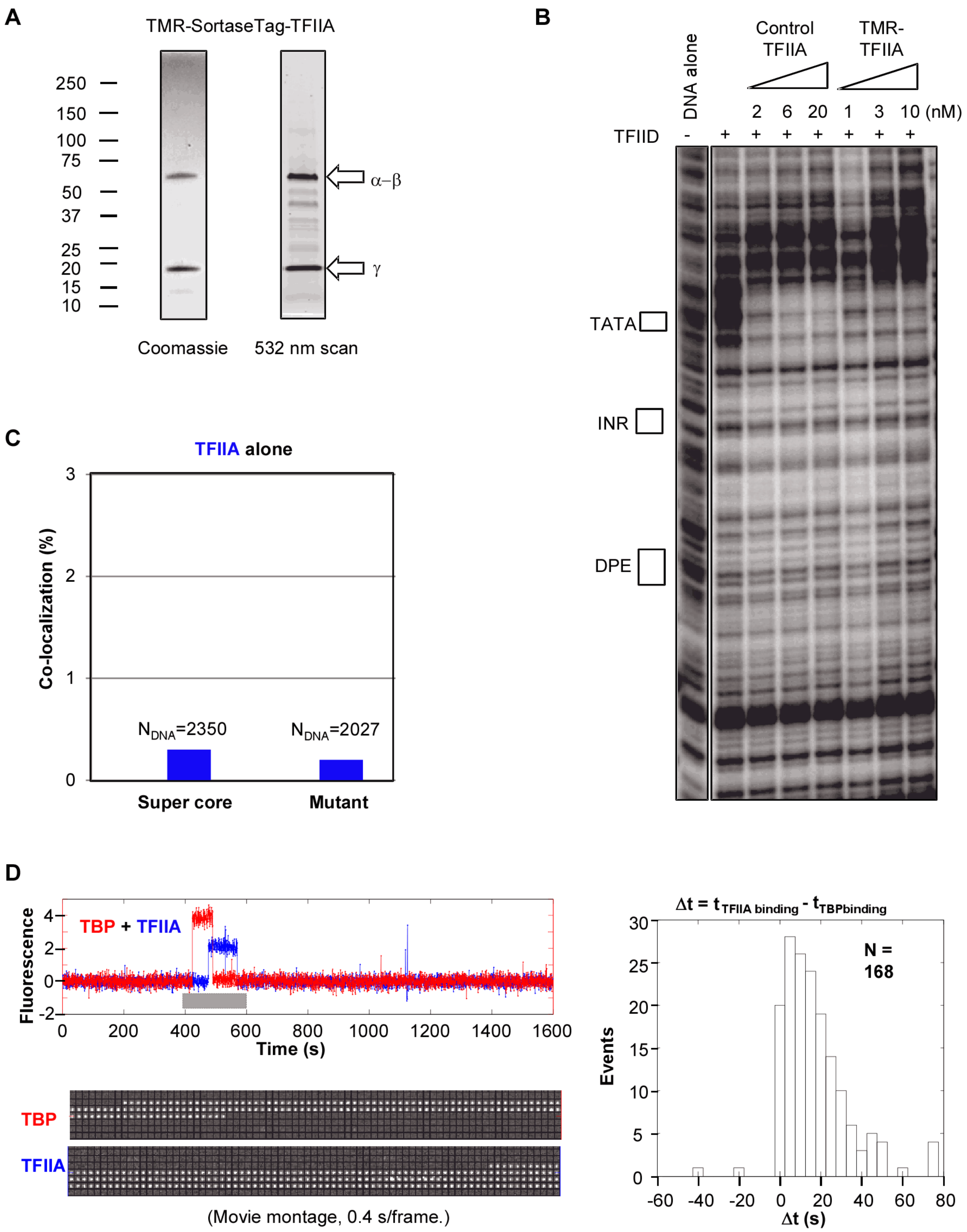
A
TMR-SortaseTag-TFIIA
250
150
100
75
50
37
25
20
15
10
α–β
γ
Coomassie
532 nm scan
B
DNA alone
Control TFIIA
TMR-TFIIA
2 6 20 1 3 10 (nM)
TFIID - + + + + + + +
TATA
INR
DPE
C
TFIIA alone
Co-localization (%)
N_DNA=2350
N_DNA=2027
Super core
Mutant
D
TBP + TFIIA
Fluorescence
Time (s)
TBP
TFIIA
(Movie montage, 0.4 s/frame.)
Δt = t_TFIIA binding - t_TBPbinding
N = 168
Events
Δt (s)

Zhang_FigS5

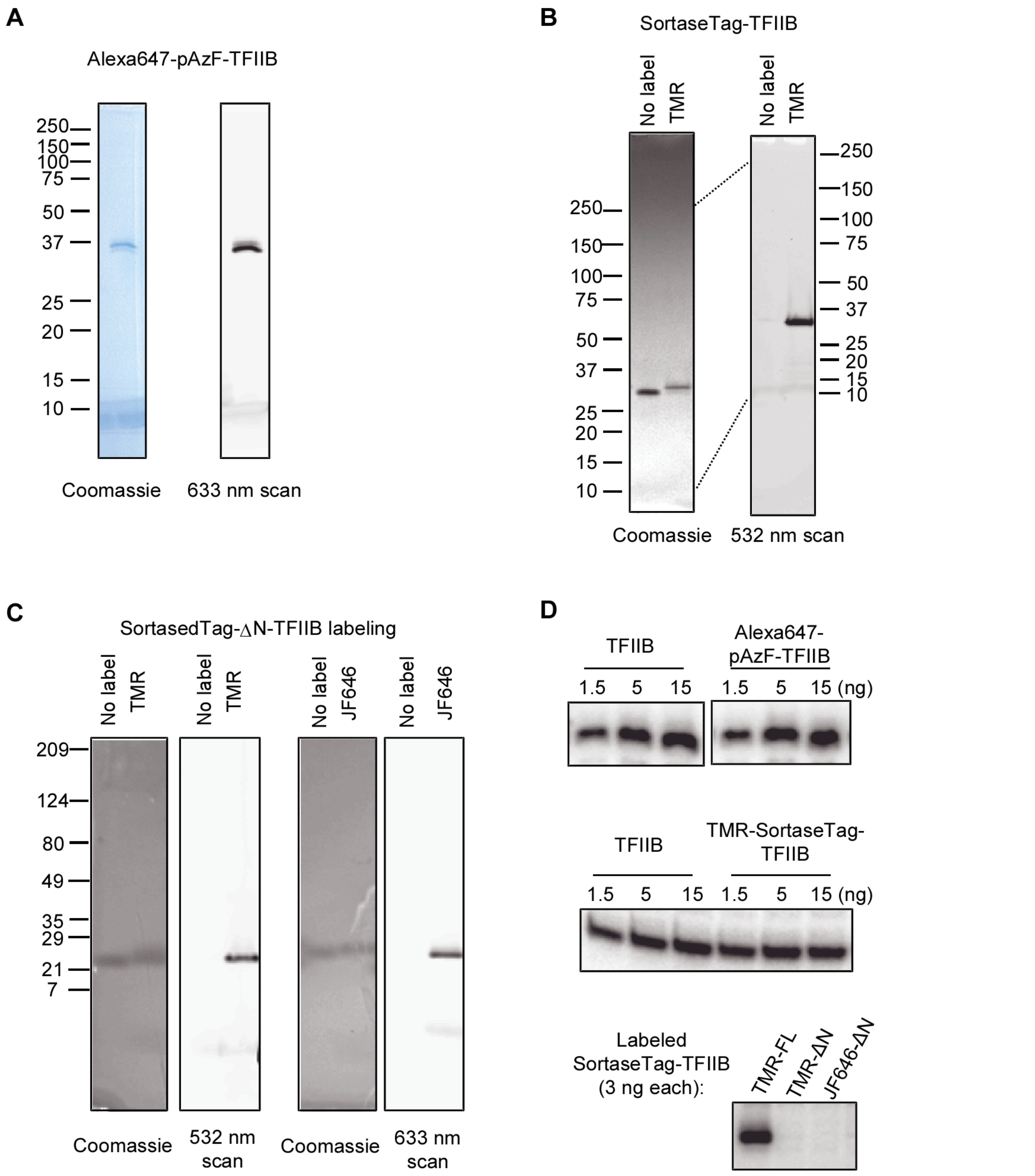
A
Alexa647-pAzF-TFIIB
250
150
100
75
50
37
25
20
15
10
Coomassie
633 nm scan
B
SortaseTag-TFIIB
No label
TMR
No label
TMR
250
150
100
75
50
37
25
20
15
10
Coomassie
532 nm scan
C
SortasedTag-∆N-TFIIB labeling
No label
TMR
No label
TMR
No label
JF646
No label
JF646
209
124
80
49
35
29
21
7
Coomassie
532 nm scan
Coomassie
633 nm scan
D
TFIIB
Alexa647-pAzF-TFIIB
1.5 5 15 1.5 5 15 (ng)
TFIIB
TMR-SortaseTag-TFIIB
1.5 5 15 1.5 5 15 (ng)
Labeled SortaseTag-TFIIB (3 ng each):
TMR-FL
TMR-∆N
JF646-∆N

Zhang_Fig S5 (continued)

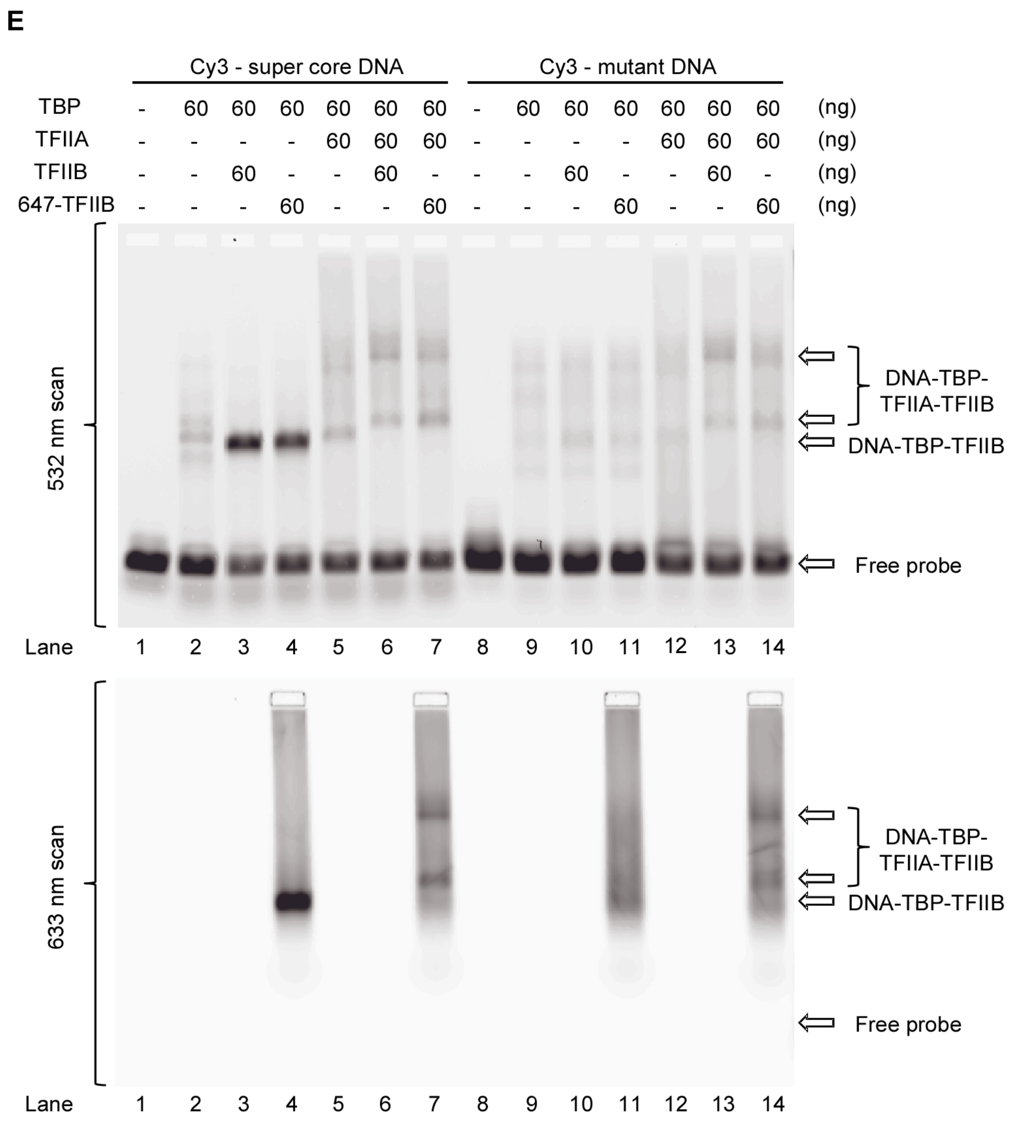

Zhang_Fig S5 (continued)

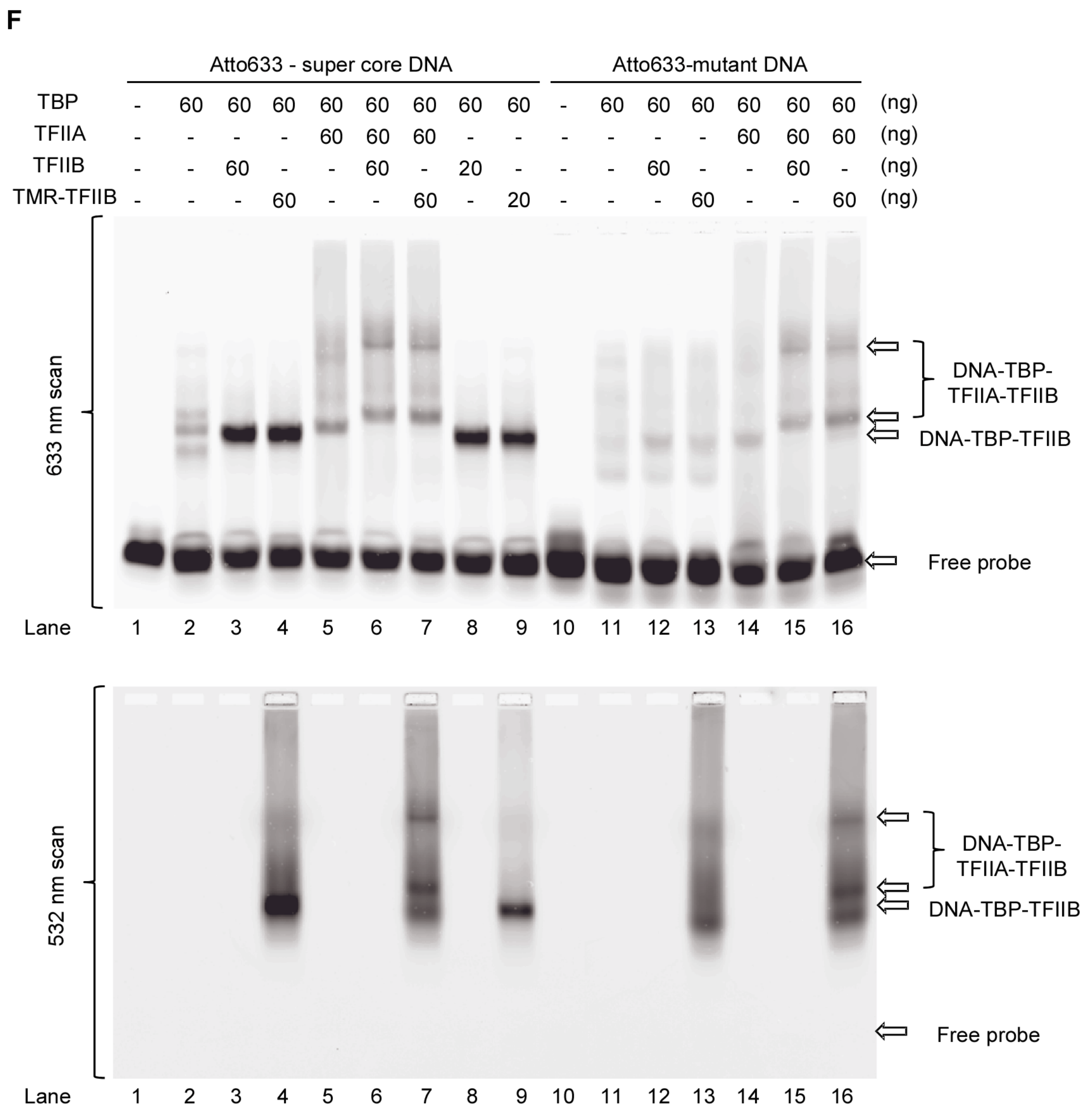

Zhang_Fig S5 (continued)

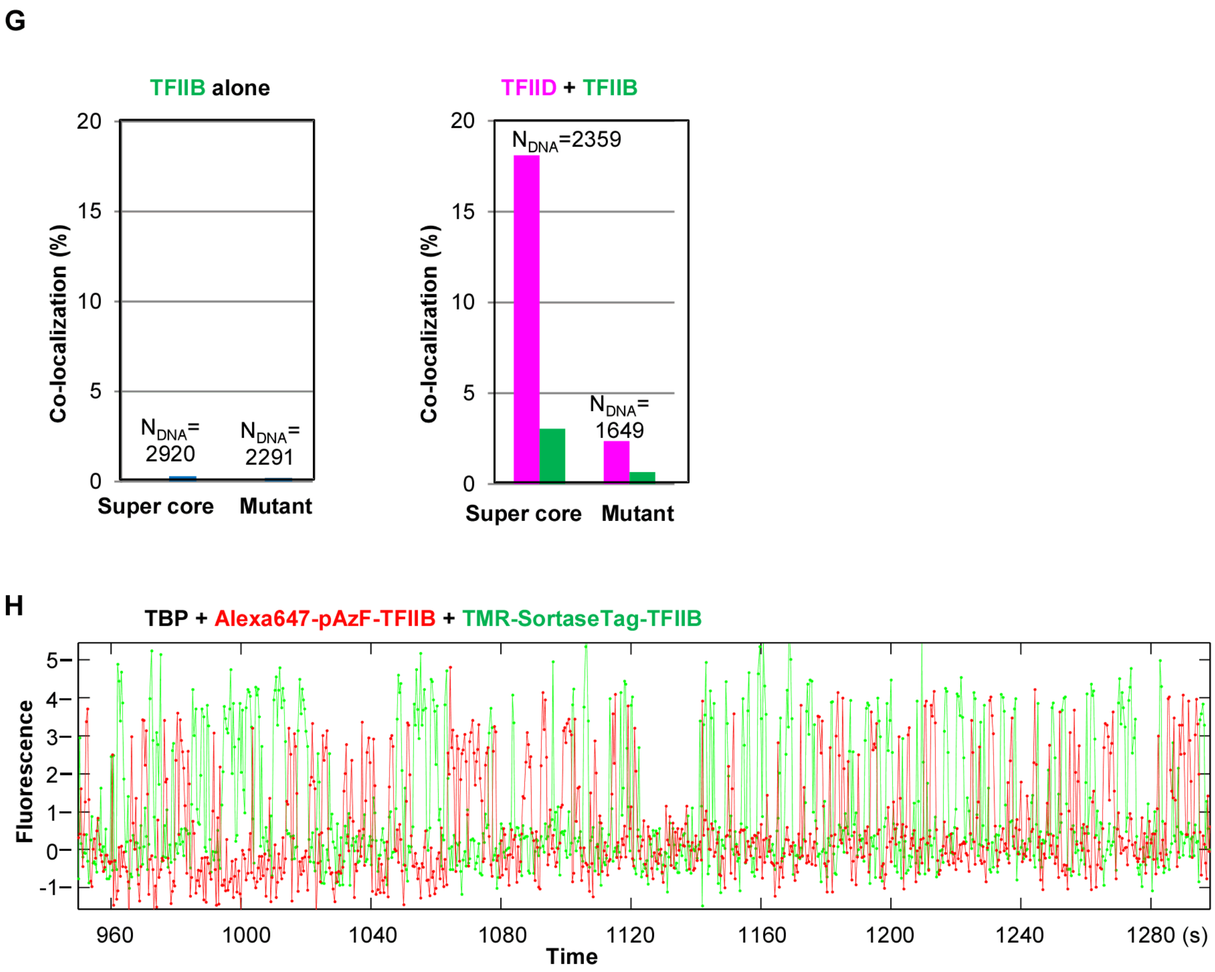

Zhang_Fig S6

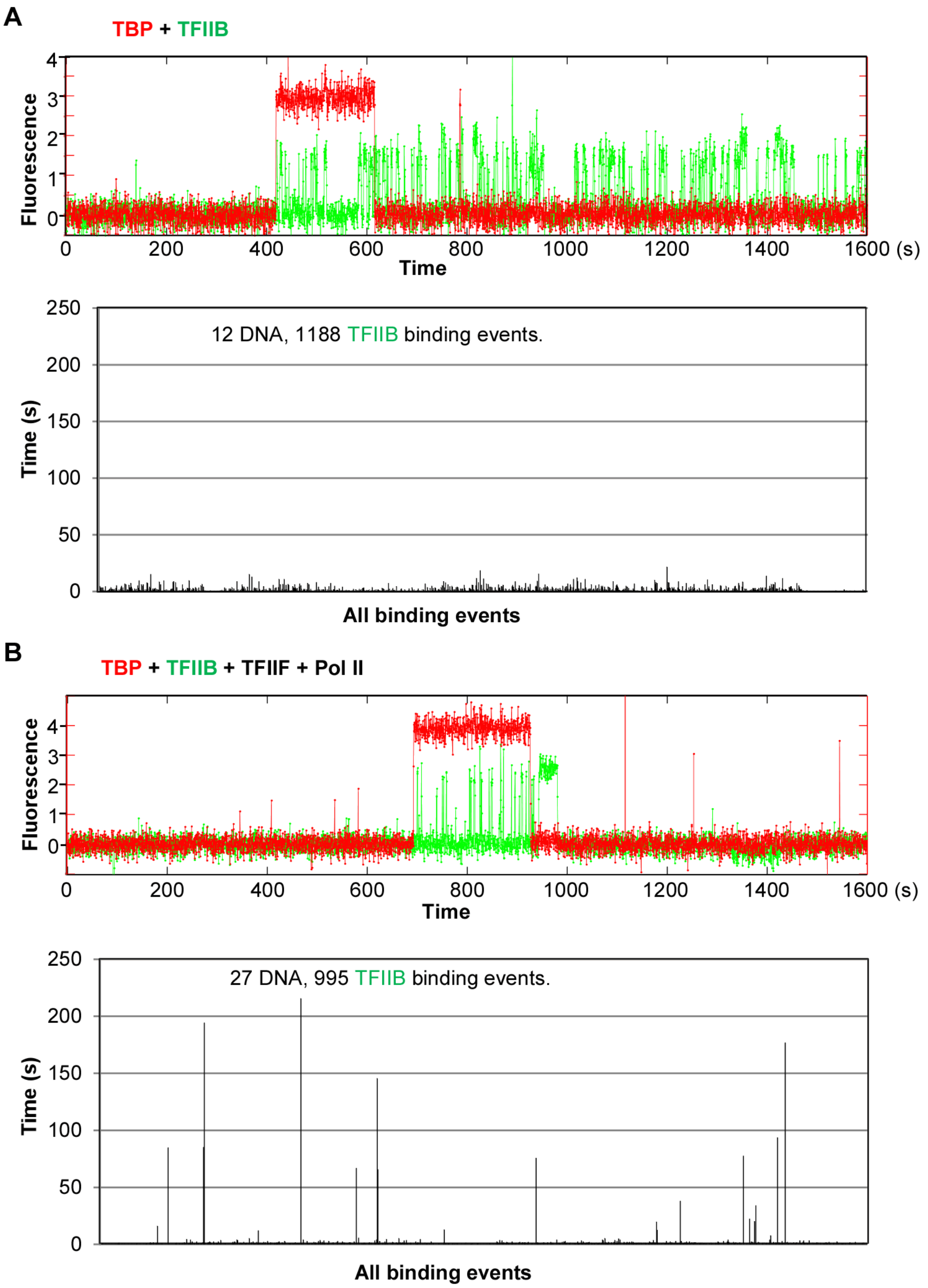
A
TBP + TFIIB
Fluorescence
Time
0 200 400 600 800 1000 1200 1400 1600 (s)
Time (s)
12 DNA, 1188 TFIIB binding events.
All binding events
B
TBP + TFIIB + TFIIF + Pol II
Fluorescence
Time
0 200 400 600 800 1000 1200 1400 1600 (s)
Time (s)
27 DNA, 995 TFIIB binding events.
All binding events

Zhang_Fig S7

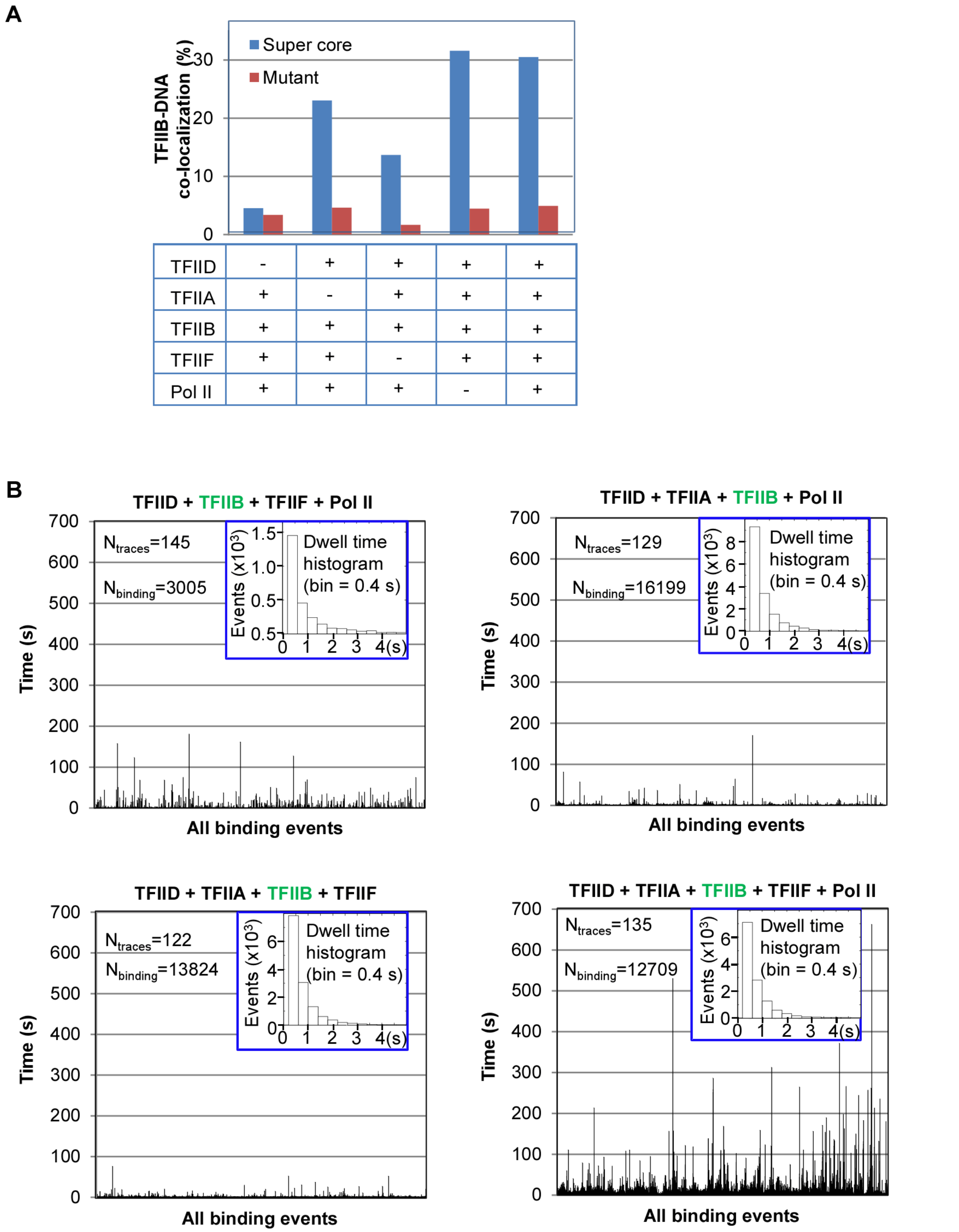
A
Super core
Mutant
TFIIB-DNA co-localization (%)
0
10
20
30
TFIID - + + + +
TFIIA + - + + +
TFIIB + + + + +
TFIIF + + - + +
Pol II + + + - +
B
TFIID + TFIIB + TFIIF + Pol II
Ntraces=145
Nbinding=3005
Dwell time histogram (bin = 0.4 s)
Events (x10³)
Time (s)
All binding events
TFIID + TFIIA + TFIIB + Pol II
Ntraces=129
Nbinding=16199
TFIID + TFIIA + TFIIB + TFIIF
Ntraces=122
Nbinding=13824
TFIID + TFIIA + TFIIB + TFIIF + Pol II
Ntraces=135
Nbinding=12709

Zhang_Fig S8

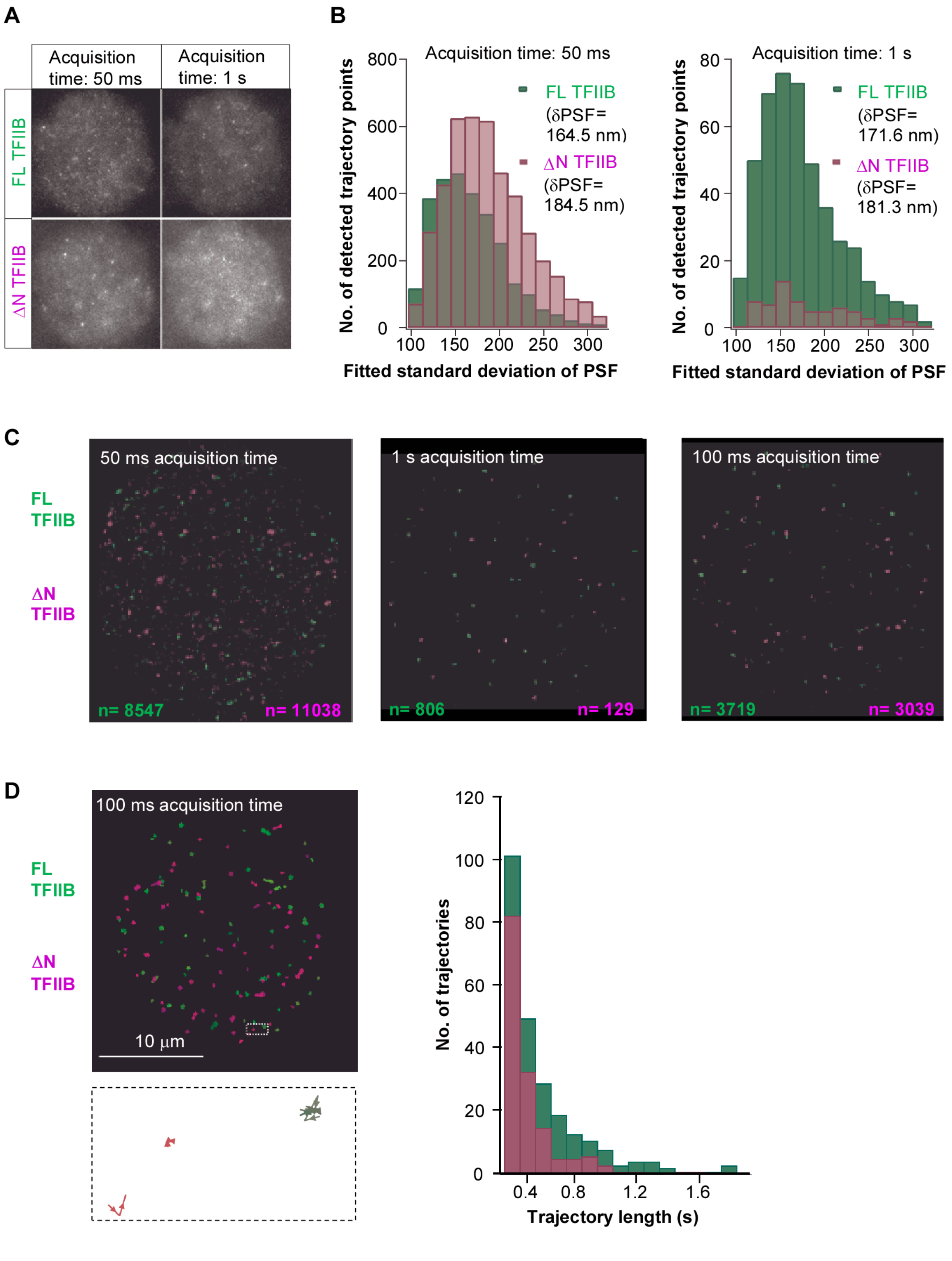
A
Acquisition time: 50 ms
Acquisition time: 1 s
FL TFIIB
ΔN TFIIB
B
Acquisition time: 50 ms
FL TFIIB (δPSF= 164.5 nm)
ΔN TFIIB (δPSF= 184.5 nm)
No. of detected trajectory points
Fitted standard deviation of PSF
Acquisition time: 1 s
FL TFIIB (δPSF= 171.6 nm)
ΔN TFIIB (δPSF= 181.3 nm)
No. of detected trajectory points
Fitted standard deviation of PSF
C
FL TFIIB
ΔN TFIIB
50 ms acquisition time
n= 8547
n= 11038
1 s acquisition time
n= 806
n= 129
100 ms acquisition time
n= 3719
n= 3039
D
FL TFIIB
ΔN TFIIB
100 ms acquisition time
10 μm
No. of trajectories
Trajectory length (s)

Supplemental Methods: protein labeling

Human TFIID labeling was designed using a non-covalent binding of a histidine-rich region within the C-terminus of the TAF2 subunit (amino acid residues 1146-1171, sequence: HHHHHHHEHKKKKKKHKHKHKHKHKH) that is intrinsically disordered, thus structurally accessible (Zhang et al., 2015), by an Atto565-conjugated tris-nitrilotriacetic acid compound coordinated with Ni^{2+} (Atto565-Tris-NTA, Figure S1A, a gift from Piehler Jacob) (Strunk et al., 2009). Purified TFIID ~100 nM was mixed with 500 nM Atto565-Tris-NTA in a buffer (10% glycerol, 25 mM HEPES pH 7.9, 12.5 mM $MgCl_2$, 100 mM KCl, 0.1 mM EDTA, 0.01 % NP40 and 1 mM DTT), incubated on ice for 3 h followed by room temperature for 3 min, then diluted 100 fold for single-molecule imaging (final concentration of ~1 nM for TFIID and 5 nM for the Atto565-Tris-NTA). With an multiple nitrilotriacetic acid moieties coordinated with Ni^{2+} on a circular scaffold, Atto565-Tris-NTA has the potential to bind poly histidine stretches with sub nanomolar affinity (Lata et al., 2005). Its specific recognition of the TAF2 subunit of the TFIID complex was confirmed by staining an SDS-PAGE gel (overnight staining followed by overnight de-staining in water, and scanned by Typhoon Trio+ imager (GE Healthcare) (Figure S2B). The same Atto565-Tris-NTA (1 µM) was added to a standard transcription assay and was found to have no effect on transcription activity (Figure S2C). We had previously reported that a tin(IV) oxochloride derived metal cluster targeting overlapping histidine-rich region specifically arrests TFIID-directed transcription initiation (Zhang et al., 2015). The structural flexibility of the Tris-NTA compound might be the reason that prevents it from affecting TFIID function, despite the

tight binding. This strategy of labeling can detect ~80-90% of the TFIID binding events based on two color single-molecule experiments using Alexa647 labeled TFIIB in the presence of TFIIA, and in which a TFIID binding event is expected to occur prior to binding by TFIIB.

Recombinant human TBP, fused to a 6xHis tag followed by a HaloTag at the N-terminus, was purified by the standard procedure using Ni-NTA resin, and further cleaned up by a heparin column (loaded in 50 mM HEPES pH 7.9, 150 mM NaCl, 1 mM DTT; washed with 20 mM HEPES pH 7.9, 150 mM KCl, 1 mM DTT, 0.5 mM EDTA and 10% glycerol; and eluted with a KCl gradient from 150 mM to 1 M, with peak fraction collected at ~ 0.3 M KCl). For labeling, ~ 10 uM purified protein was mixed with 30 uM Halo-JF549 or Halo-JF646 ligands (with 2% DMSO) and incubated at 23°C for 10 min. Free ligands were removed by Zeba Spin desalting columns (ThermoFisher). Labeling efficiency was determined to be >80% by single-molecule experiments (in two-color TBP and TFIIB binding assays, >80% of repetitive TFIIB binding was proceeded by a TBP signal).

The sortase-mediated fluorescent labeling of TFIIA and TFIIB was as previously described (with an N-terminal GST-SUMO-GGGG tag) with minor modifications (Ticau et al., 2015). For TFIIA, the tag was inserted at the N-terminus of the α-β fusion subunit in an ampicillin-resistant vector, co-expressed with a FLAG tagged γ subunit (with a sequence of MGSDYKDDDDKG added to the N-terminus: the FLAG tag is underscored, the remaining amino acid residues are from a poly-cloning site) in a kanamycin-resistant vector. When expressed in E. coli, the first methionine (M) gets removed by the host cell

thus exposing a glycine (G) residue, making the γ subunit a substrate for sortase (Figure S3A). A GST fusion of TFIIA (complex of α-β fusion and the γ subunit) was expressed and purified from *E. coli* Rosetta cells, and digested by 0.5 µg/ml Ulp1 (His-tagged, in-house prepared) and mixed with 50% glutathione-crosslinked beads slurry in buffer (20 mM Tris-HCl pH 8.0 and 500 mM NaCl) for ~3-10 min at room temperature. Released proteins were immediately further purified by a Superdex 200 10/300GL size-exclusion column (GE Healthcare). The labeling reaction was carried out at room temperature for 1 hr with 5 µM TFIIA, 5 µM sortase (His-tagged, in-house preparation), 800 µM 5-carboxytetramethylrhodamine conjugated LPETGG peptide (Innovagen SP-5364-1), 5 mM $CaCl_2$ and 5 mM $MgCl_2$. The His-tagged sortase was removed by mixing with 1/10 volume of Ni-NTA agarose beads at 4C for 5 min. The reaction mixture was further purified by Superdex 200 10/300GL column to remove free dyes in buffer (10% glycerol, 20 mM Tris HCl pH 8.0, 500 mM NaCl, and 1 mM DTT). Samples were taken during the labeling reaction, separated by SDS-PAGE and scanned at 532 nm to monitor the reaction. The α-β subunit (with an optimal GGGG sequence at the N-terminus) completed the reaction within ~5 min, while the γ subunit (with a non-optimal G residue) completed the reaction in 30 min. This dual target situation led to a high labeling efficiency. To test the labeling efficiency, TFIIA was incubated together with Alexa647 labeled TFIIB and unlabeled TFIID for single-molecule promoter binding analysis. ~95% of the rapid, repetitive TFIIB binding was found to be proceeded by a TFIIA binding signal, suggesting that ~95% of the TFIIA molecules have at least one fluorophore.

Sortase-mediate labeling of TFIIB (full-length or Δ1-106 mutant) was carried out in the same way as TFIIA labeling, except that the Ulp1 digestion and subsequent size-exclusion chromatography was carried out in a different buffer (20 mM HEPES ph7.6, 300 mM KCl, 20 µM ZnCl2, 1 mM DTT, 10% glycerol). The concentration of TFIIB and sortase were both 30 µM, and an in-house preparation of JF646-LPETGG peptide conjugate was used. The reaction was diluted to 250 mM KCl with a matching buffer, then loaded onto a 1 mL HiTrap Heparin HP column (GE Healthcare 17-0406-01) and eluted with a salt gradient of 0.25-1 M KCl (peak elution at about 0.5~0.6 M). The unreacted peptide ligand was in the flow-through fraction.

Full-length TFIIB was also labeled using unnatural amino acid mediated conjugation (Kim et al., 2013). In brief, a sequence of MGS(pAzF)SHHHHHH SSGLVPRGSH (pAzF stands for para-azidyl phenylalanine, the rest are linkers including a thrombin cleavage site from the vector) was attached to the N-terminus of human TFIIB and purified by Ni-NTA affinity resin (final buffer: 20 mM Tris-HC1 pH 7.9 at 4°, 10% glycerol, 1 mM DTT, 0.2 mM EDTA, 0.5 mM PMSF, and 300 mM KC1). 18 µM protein was mixed with 100 µM Click-IT® Alexa Fluor® 647 DIBO Alkyne (Invitrogen C10408) and incubated at room temperature for 3 hours. This usually allows 50% of the proteins to be labeled which will appear as a slightly upper shifted band when separated by 12% SDS-PAGE gel. The reaction was passed through a PD10 desalting column by gravity to remove the unreacted dye and exchanged into a low salt buffer (10% glycerol, 25 mM HEPES pH 7.9, 12.5 mM $MgCl_2$, 200 mM KCl, 0.1 mM EDTA, and 1 mM DTT). The peak fractions were collected and loaded onto a Poros® Heparin column

(Applied Biosciences 4333413). A 0.2-1 M KCl salt gradient allows elution and moderate separation of the labeled species (eluted at lower salt) from the unlabeled species. The fraction used for single-molecule reaction contains ~90% labeled species.

JF646-LPETGG peptide conjugate synthesis

Commercial reagents were obtained from reputable suppliers and used as received. All solvents were purchased in septum-sealed bottles stored under an inert atmosphere. Reactions were monitored by thin layer chromatography (TLC) on pre-coated TLC glass plates (silica gel 60 F_{254}, 250 µm thickness) or by LC/MS (4.6 mm × 150 mm 5 µm C18 column; 5 µL injection; 10–95% MeCN/H_2O, linear gradient, with constant 0.1% v/v TFA additive; 20 min run; 1 mL/min flow; ESI; positive ion mode; UV detection at 650 nm). Mass spectrometry was performed by the High Resolution Mass Spectrometry Facility at the University of Iowa. NMR spectra were recorded on a 400 MHz spectrometer. ^{1}H and ^{13}C chemical shifts (δ) were referenced to TMS or residual solvent peaks. Data for ^{1}H NMR spectra are reported as follows: chemical shift (δ ppm), multiplicity (s = singlet, d = doublet, t = triplet, q = quartet, dd = doublet of doublets, m = multiplet), coupling constant (Hz), integration.

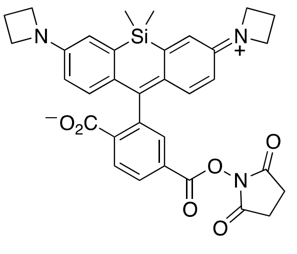

JF$_{646}$-NHS: 6-Carboxy-JF$_{646}$ (Grimm et al., 2015) (40 mg, 65.5 µmol, TFA salt) was combined with *N,N′*-disuccinimidyl carbonate (37 mg, 144 µmol, 2.2 eq) in DMF (2.5 mL). After adding triethylamine (55 µL, 393 µmol, 6 eq) and 4-(dimethylamino)pyridine (0.8 mg, 6.55 µmol, 0.1 eq), the reaction was stirred at room temperature for 3 h. It was subsequently diluted with 10% w/v citric acid and extracted with EtOAc (2×). The combined organic extracts were washed with brine, dried over anhydrous $MgSO_4$, filtered, and concentrated *in vacuo*. Flash chromatography (0–50% EtOAc/toluene, linear gradient) yielded 31 mg (80%) of the title compound as a yellow-green solid. ^{1}H NMR ($CDCl_3$, 400 MHz) δ 8.27 (dd, *J* = 8.0, 1.4 Hz, 1H), 8.07 (dd, *J* = 8.0, 0.7 Hz, 1H), 8.00 (dd, *J* = 1.3, 0.7 Hz, 1H), 6.74 (d, *J* = 8.7 Hz, 2H), 6.66 (d, *J* = 2.6 Hz, 2H), 6.30 (dd, *J* = 8.7, 2.7 Hz, 2H), 3.91 (t, *J* = 7.3 Hz, 8H), 2.89 (s, 4H), 2.37 (p, *J* = 7.2 Hz, 4H), 0.62 (s, 3H), 0.56 (s, 3H); ^{13}C NMR ($CDCl_3$, 101 MHz) δ 169.4 (C), 169.0 (C), 161.1 (C), 155.5 (C), 151.2 (C), 136.5 (C), 131.7 (C), 131.6 (C), 130.7 (CH), 130.1 (C), 127.8 (CH), 126.8 (CH), 126.3 (CH), 115.8 (CH), 112.7 (CH), 92.4 (C), 52.3 (CH_2), 25.8 (CH_2), 17.0 (CH_2), 0.3 (CH_3), -1.1 (CH_3); HRMS (ESI) calcd for $C_{33}H_{32}N_3O_6Si$ $[M+H]^+$ 594.2055, found 594.2069.

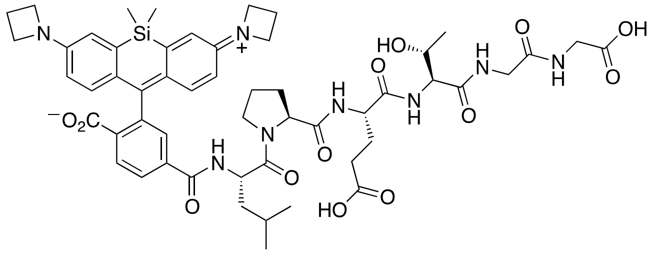

JF$_{646}$-LPETGG: JF$_{646}$-NHS (2.0 mg, 3.37 µmol) and Leu-Pro-Glu-Thr-Gly-Gly (3.5 mg, 5.05 µmol, 1.5 eq) were combined in DMF (1 mL), and *N,N*-

diisopropylethylamine (2.9 µL, 16.8 µmol, 5 eq) was added. The reaction was stirred at room temperature for 18 h. It was subsequently concentrated *in vacuo* and purified by reverse phase HPLC (10–75% MeCN/H_2O, linear gradient, with constant 0.1% v/v TFA additive) to provide 2.9 mg (74%, TFA salt) of the title compound as a blue solid. Analytical HPLC: t_R = 11.1 min, >99% purity (4.6 mm × 150 mm 5 µm C18 column; 5 µL injection; 10–95% MeCN/H_2O, linear gradient, with constant 0.1% v/v TFA additive; 20 min run; 1 mL/min flow; ESI; positive ion mode; detection at 650 nm); MS (ESI) calcd for $C_{53}H_{67}N_8O_{13}Si$ $[M+H]^+$ 1051.5, found 1051.3.

References

GRIMM JB, ENGLISH BP, CHEN J, SLAUGHTER JP, ZHANG Z, REVYAKIN A, PATEL R, MACKLIN JJ, NORMANNO D, SINGER RH, et al. 2015. A general method to improve fluorophores for live-cell and single-molecule microscopy. *Nat Methods* **12:** 244-250, 3 p following 250.

HUANG B, BABCOCK H, ZHUANG X. 2010. Breaking the diffraction barrier: super-resolution imaging of cells. *Cell* **143:** 1047-1058.

KIM CH, AXUP JY, SCHULTZ PG. 2013. Protein conjugation with genetically encoded unnatural amino acids. *Curr Opin Chem Biol* **17:** 412-419.

LATA S, REICHEL A, BROCK R, TAMPE R, PIEHLER J. 2005. High-affinity adaptors for switchable recognition of histidine-tagged proteins. *J Am Chem Soc* 127: 10205-10215.

PRESOLSKI SI, HONG VP, FINN MG. 2011. Copper-Catalyzed Azide-Alkyne Click Chemistry for Bioconjugation. *Curr Protoc Chem Biol* **3:** 153-162.

REVYAKIN A, ZHANG Z, COLEMAN RA, LI Y, INOUYE C, LUCAS JK, PARK SR, CHU S, TJIAN R. 2012. Transcription initiation by human RNA polymerase II visualized at single-molecule resolution. *Genes Dev* **26:** 1691-1702.

STRUNK JJ, GREGOR I, BECKER Y, LAMKEN P, LATA S, REICHEL A, ENDERLEIN J, PIEHLER J. 2009. Probing protein conformations by in situ non-covalent fluorescence labeling. *Bioconjug Chem* **20:** 41-46.

TICAU S, FRIEDMAN LJ, IVICA NA, GELLES J, BELL SP. 2015. Single-molecule studies of origin licensing reveal mechanisms ensuring bidirectional helicase loading. *Cell* **161:** 513-525.

ZHANG Z, BOSKOVIC Z, HUSSAIN MM, HU W, INOUYE C, KIM HJ, ABOLE AK, DOUD MK, LEWIS TA, KOEHLER AN, et al. 2015. Chemical perturbation of an intrinsically disordered region of TFIID distinguishes two modes of transcription initiation. *Elife,* **4**.
